# Targeting the microglia-synapse interface in depression: molecular mechanisms and pharmacological perspectives from traditional Chinese medicine

**DOI:** 10.3389/fimmu.2026.1972377

**Published:** 2026-10-26

**Authors:** Shu-xia Yao, Zi-yu Xu, Hong-qing Zhao, Dong-fang Deng, Yi Yang, Lin Tang, Hu Tan, Jian Liu

**Affiliations:** 1 The First Hospital, Hunan University of Chinese Medicine, Changsha, Hunan, China; 2 Academy of Chinese Medical Sciences, Hunan University of Chinese Medicine, Changsha, Hunan, China; 3 Hunan Key Laboratory of Traditional Chinese Medicine Prevention and Treatment of Depressive Diseases, Changsha, Hunan, China

**Keywords:** depression, microglia, molecular mechanisms, synaptic pruning, traditional Chinese medicine

## Abstract

Depression is a prevalent neuropsychiatric disorder characterized by impaired synaptic plasticity, disrupted neural circuits, and persistent neuroimmune dysregulation. Increasing evidence indicates that microglia-mediated synaptic pruning, a physiological process essential for neural circuit refinement, becomes abnormally activated under depressive conditions, leading to excessive synaptic elimination and impaired connectivity. Complement-dependent pathways, inflammatory signaling, mitochondrial dysfunction, and alterations in the neural microenvironment contribute to microglial activation and synaptic remodeling. Traditional Chinese Medicine (TCM), with its multi-component and multi-target properties, has shown potential antidepressant effects by regulating neuroinflammation, immune homeostasis, and synaptic plasticity. This review summarizes recent advances in microglia-mediated synaptic pruning in depression and discusses the potential role of TCM interventions in this neuroimmune pathway. Current studies suggest that stress-induced microenvironmental changes promote microglial activation and excessive synaptic engulfment, while TCM formulas, herbal extracts, and bioactive compounds may restore synaptic function by modulating microglial polarization, inflammatory responses, complement signaling, and mitochondrial homeostasis. However, direct evidence demonstrating the regulation of microglia-mediated synaptic pruning by TCM remains limited. Future research integrating single-cell sequencing, advanced imaging, and molecular tracing approaches is required to clarify these mechanisms. Overall, targeting microglial synaptic remodeling provides a promising framework for understanding depression pathogenesis and developing novel TCM-based therapeutic strategies.

## Graphical Abstract

**Figure:**
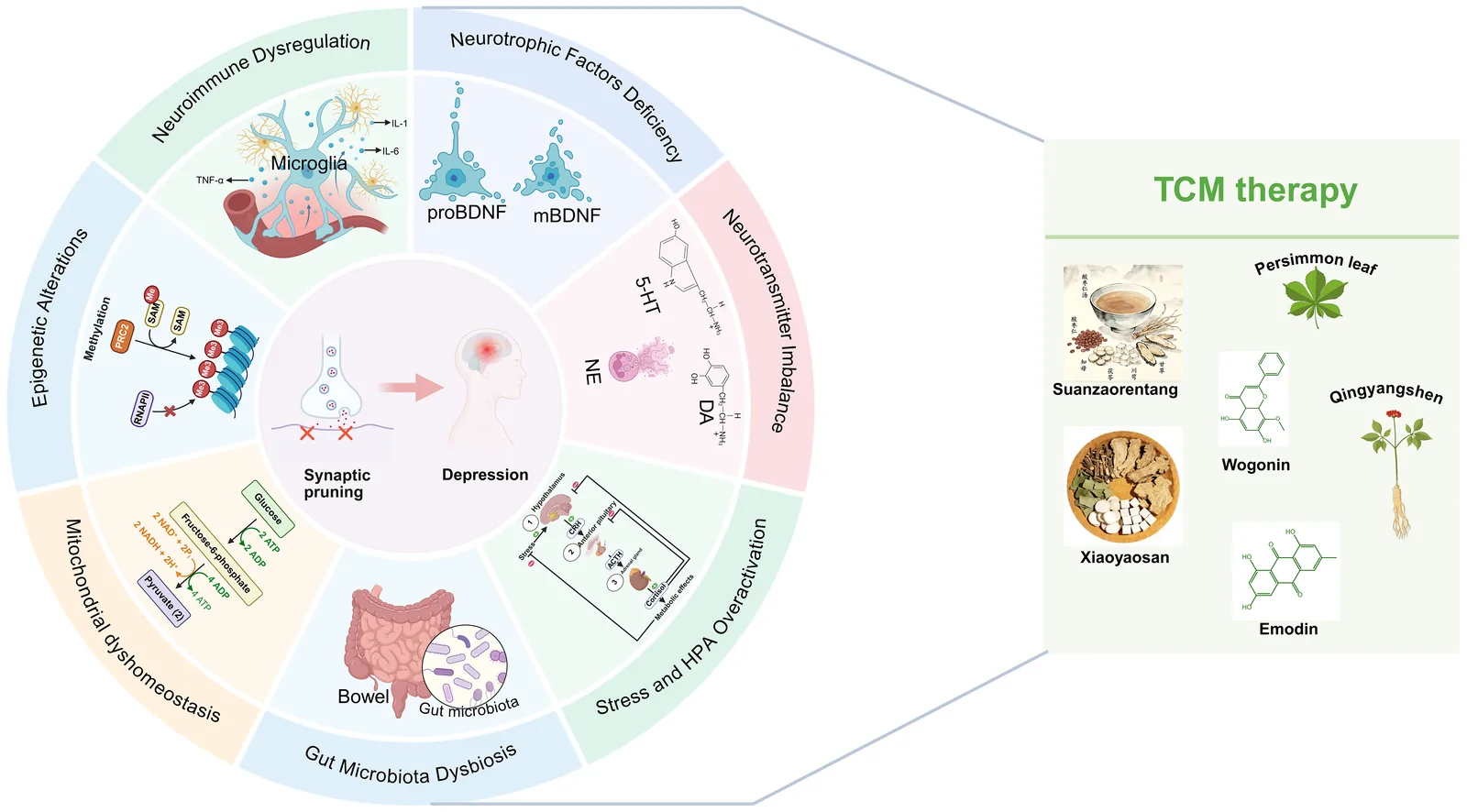
This schematic summarizes seven major mechanisms linking dysregulated synaptic pruning to depression. Representative TCM formulas and bioactive compounds may improve synaptic plasticity through regulation of neuroinflammation, neurotrophic signaling, neurotransmission, and related pathways. Direct evidence demonstrating that TCM interventions regulate microglial synaptic engulfment remains limited.

## 1 Introduction

According to the World Health Organization (WHO), depression affects approximately 350 million people worldwide and remains one of the leading causes of disability (1). Current therapeutic strategies primarily encompass antidepressant medications, psychotherapy, and non-pharmacological interventions. However, conventional antidepressants are frequently limited by delayed onset of action, modest efficacy, adverse effects, and high relapse rates. Consequently, elucidating novel pathogenic mechanisms and identifying new therapeutic targets for depression hold significant scientific value and clinical importance. Recent studies have indicated that excessive synaptic pruning within the hippocampus contributes to excitatory synapse loss, impaired circuit connectivity, and abnormal information processing, thereby promoting depressive-like behaviors (2). Increasing evidence demonstrates that abnormalities in synaptic plasticity, manifested as reduced dendritic spine density, decreased expression of synaptic proteins, and impaired long-term potentiation (LTP), are present in emotion-regulating brain regions such as the prefrontal cortex and hippocampus of both depressed patients and animal models (3, 4). These alterations do not occur in isolation; rather, they are intricately intertwined with multiple mechanisms, including neurotrophic factor deficiency, neuroimmune dysregulation, neurotransmitter imbalance, hypothalamic-pituitary-adrenal (HPA) axis dysfunction, gut microbiota dysbiosis, mitochondrial homeostasis imbalance, and epigenetic modifications. Collectively, these factors contribute to the onset and progression of depression.

Within the dynamic regulatory network of synaptic plasticity, microglia, as the resident immune cells of the central nervous system, play a pivotal role. Recent research has revealed that microglia continuously survey the synaptic microenvironment under physiological conditions. They regulate neural circuits by recognizing and engulfing redundant or weakened synaptic structures, a process termed “synaptic pruning” (5). During development, microglia utilize complement-dependent mechanisms (e.g., C1q/C3 signaling) to eliminate excessive or weak synaptic connections; in adulthood, they shift to a homeostatic maintenance mode, clearing aged or dysfunctional synapses. This pruning process is tightly regulated by a triad of signals: “Find Me”, “Eat Me”, and “Don’t Eat Me”. Dysregulation of these signals may disrupt the balance between synaptic elimination and preservation, thereby contributing to impaired synaptic plasticity (6). Evidence indicates that in depression models, microglial overactivation is accompanied by upregulated complement C3 and aberrant synaptic engulfment, while inhibiting microglial activity or blocking complement signaling can partially reverse synaptic loss and depressive-like behaviors (7). However, how microglia-mediated synaptic pruning specifically impacts synaptic plasticity deficits in depression, along with its complete molecular signaling network and interactions with other pathological mechanisms (such as neuroinflammation and BDNF signaling downregulation), remains to be systematically elucidated.

In recent years, accumulating evidence has suggested that TCM formulas, single herbs, and active compounds may influence synaptic plasticity and exhibit antidepressant-like effects in preclinical models through regulation of BDNF/TrkB signaling, neuroinflammatory pathways, glutamatergic transmission, and mitochondrial function. However, few reviews have systematically integrated the molecular mechanisms linking synaptic plasticity impairment in depression with emerging evidence regarding TCM regulation of microglia-mediated synaptic pruning. Therefore, this review focuses on the microglia-synapse interface as a potential convergence point linking diverse pathological processes in depression with synaptic dysfunction. We first summarize the molecular framework of microglia-mediated synaptic pruning, including “Find Me”, “Eat Me”, and “Don’t Eat Me” signaling systems, and discuss how pathological factors reshape the threshold controlling microglial recognition and elimination of synapses. We then examine how neurotrophic deficits, neuroimmune activation, neurotransmitter imbalance, HPA axis dysregulation, gut microbiota disturbance, mitochondrial dysfunction, and epigenetic alterations contribute to abnormal pruning-related synaptic vulnerability. Given the multi-component and multi-target characteristics of TCM, these interventions may provide unique opportunities to modulate neuroimmune-synaptic interactions that cannot be fully addressed by conventional single-target approaches. Finally, we summarize current evidence from TCM formulas, single herbs, and active compounds that may modulate pruning-associated pathways, while critically discussing the limitations of existing evidence and future research directions. Throughout this review, evidence related to the potential modulation of the microglia-synapse interface by TCM interventions is categorized into three levels: direct evidence, involving direct investigation of microglia-mediated synaptic pruning processes, including microglial synaptic engulfment, phagocytic activity, or mechanistic evidence directly linking microglial pruning-related pathways to synaptic elimination or preservation; associative evidence, involving alterations in pathways closely related to microglia-mediated synaptic remodeling, such as complement signaling, the CX3CL1/CX3CR1 axis, TREM2-related pathways, or microglial activation states, without direct demonstration of synaptic pruning events; and indirect evidence, involving improvements in synaptic plasticity, neuroinflammation, neurotrophic signaling, mitochondrial function, or dendritic spine density without direct assessment of microglia-mediated synaptic pruning.

## 2 Literature search strategy and evidence evaluation

A literature search was conducted in PubMed, Web of Science, Embase, and China National Knowledge Infrastructure (CNKI) databases up to April 2026. The search terms included combinations of “microglia”, “synaptic pruning”, “synaptic engulfment”, “microglia-synapse interaction”, “major depressive disorder”, “depression”, “Traditional Chinese Medicine”, “herbal medicine”, “Chinese medicinal formula”, “complement”, “neuroinflammation” and “bioactive compounds”. Additional relevant articles were identified by screening reference lists of selected publications.

Studies were included if they investigated the role of microglia-mediated synaptic remodeling in depression-related models, or the effects of TCM formulations or TCM-derived compounds on microglial function, neuroinflammation, synaptic plasticity, or related molecular pathways. Studies without mechanistic relevance or without sufficient experimental information were excluded.

Given that direct assessments of microglial synaptic engulfment following TCM intervention remain relatively scarce, the evidence was categorized into direct, associative, and indirect evidence according to the level of mechanistic validation.

## 3 Molecular basis and functional significance of microglia-mediated synaptic pruning

As resident immune cells of the central nervous system, microglia serve dual roles as “pruners” and “guardians” during neurodevelopment and in the maintenance of neural homeostasis. One of their core functions is synaptic pruning, a process that optimizes neural network connectivity through the recognition, engulfment, and degradation of redundant or functionally weak synapses (8). During early development, microglia exhibit an enhanced pruning phenotype, actively identifying and eliminating excessive or weakened synaptic connections. In the adult brain, microglia adopt a maintenance-oriented phenotype, continuously monitoring synaptic status to clear aged or damaged synaptic structures, thus maintaining synaptic homeostasis (9). This process is activity-dependent, ensuring that functionally important synapses are preserved. This pruning mechanism not only safeguards normal neural network connectivity but also provides critical clues for understanding neuropsychiatric disorders such as depression, schizophrenia, and Alzheimer’s disease, potentially contributing to circuit abnormalities and behavioral or cognitive impairments (10).

Disruption of microglial pruning homeostasis in either direction may impair neural circuit function. Insufficient pruning can result in the persistence of inappropriate synaptic connections, whereas excessive pruning may lead to pathological synaptic loss and reduced circuit connectivity. Failure of appropriate synaptic elimination may result in excessive retention of immature or unnecessary synaptic connections, thereby disrupting neural circuit refinement and information processing. In contrast to insufficient pruning, synaptic over-pruning occurs when microglia become hyperactive and excessively eliminate healthy or functional synapses. This pathological process disrupts established neural circuits and may contribute to cognitive and behavioral abnormalities. Therefore, microglia-mediated synaptic pruning must be maintained within a precise regulatory range. Both insufficient and excessive pruning represent pathological states of imbalance that disrupt neural circuit homeostasis and have been implicated in various neuropsychiatric disorders, including depression. The molecular regulation of microglia-mediated synaptic pruning involves a series of signaling pathways that control microglial recognition, engulfment, and elimination of synaptic structures, as illustrated in **Figures 1**, **2**.

**Figure 1:**
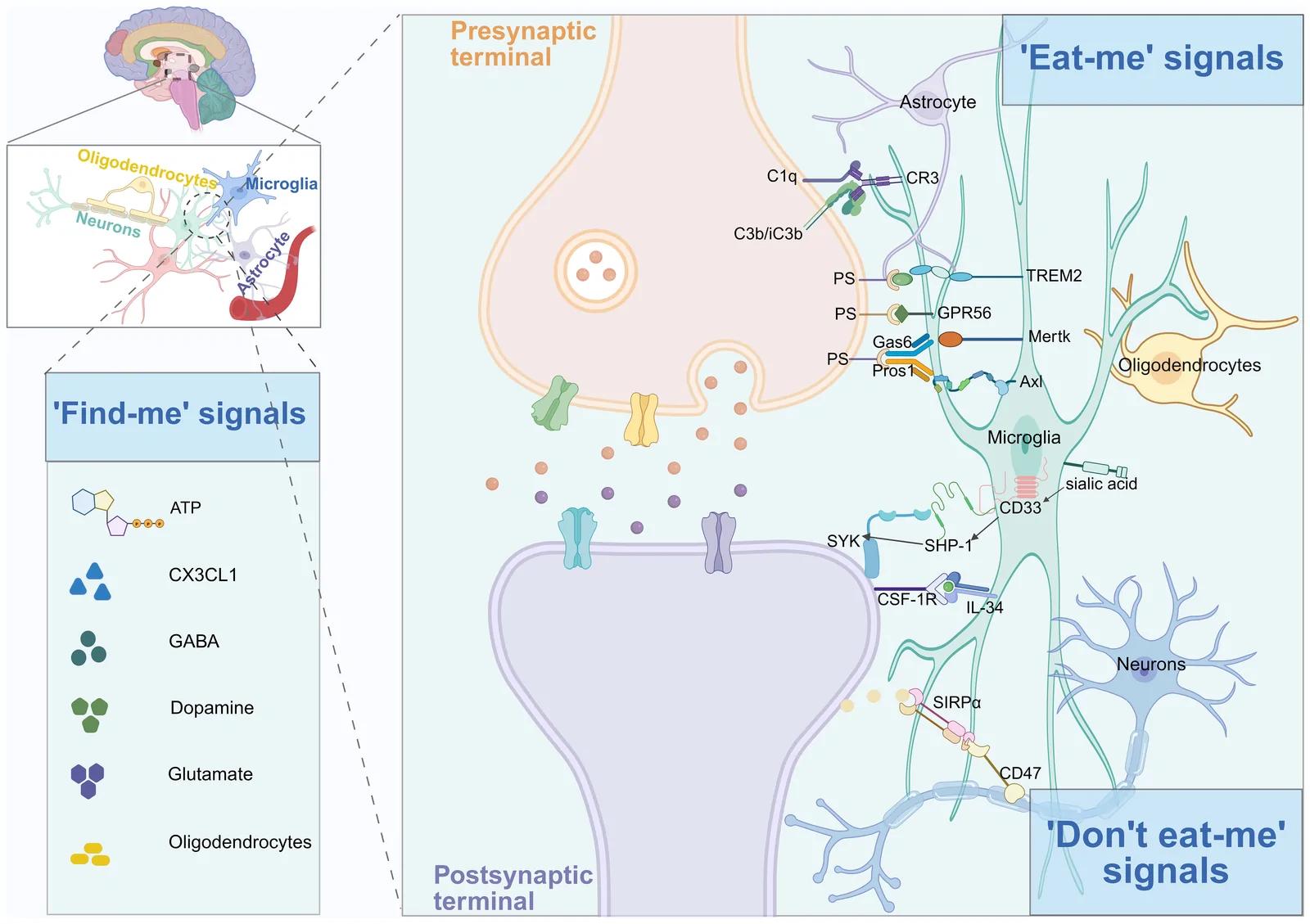
The “Find-Me”, “Eat-Me”, and “Don’t Eat-Me” signaling network in microglia-mediated synaptic pruning. This schematic illustrates the major signaling pathways involved in microglial recognition and elimination of synaptic elements. Neurons and glial cells communicate through “find-me”, “eat-me”, and “don’t eat-me” signals that regulate microglial surveillance and synaptic engulfment. Chemotactic signals, including ATP and CX3CL1, facilitate microglial interaction with synapses, whereas complement components (C1q and C3) and receptors such as CR3, TREM2, MERTK, and AXL promote synaptic recognition and elimination. In contrast, inhibitory pathways such as CD47-SIRPα prevent excessive synaptic loss. Dysregulation of these processes disrupts synaptic homeostasis and may contribute to depression-related neural circuit dysfunction. Created in BioRender. Yao, SX. (2026) https://BioRender.com/p3jgpk1.

**Figure 2:**
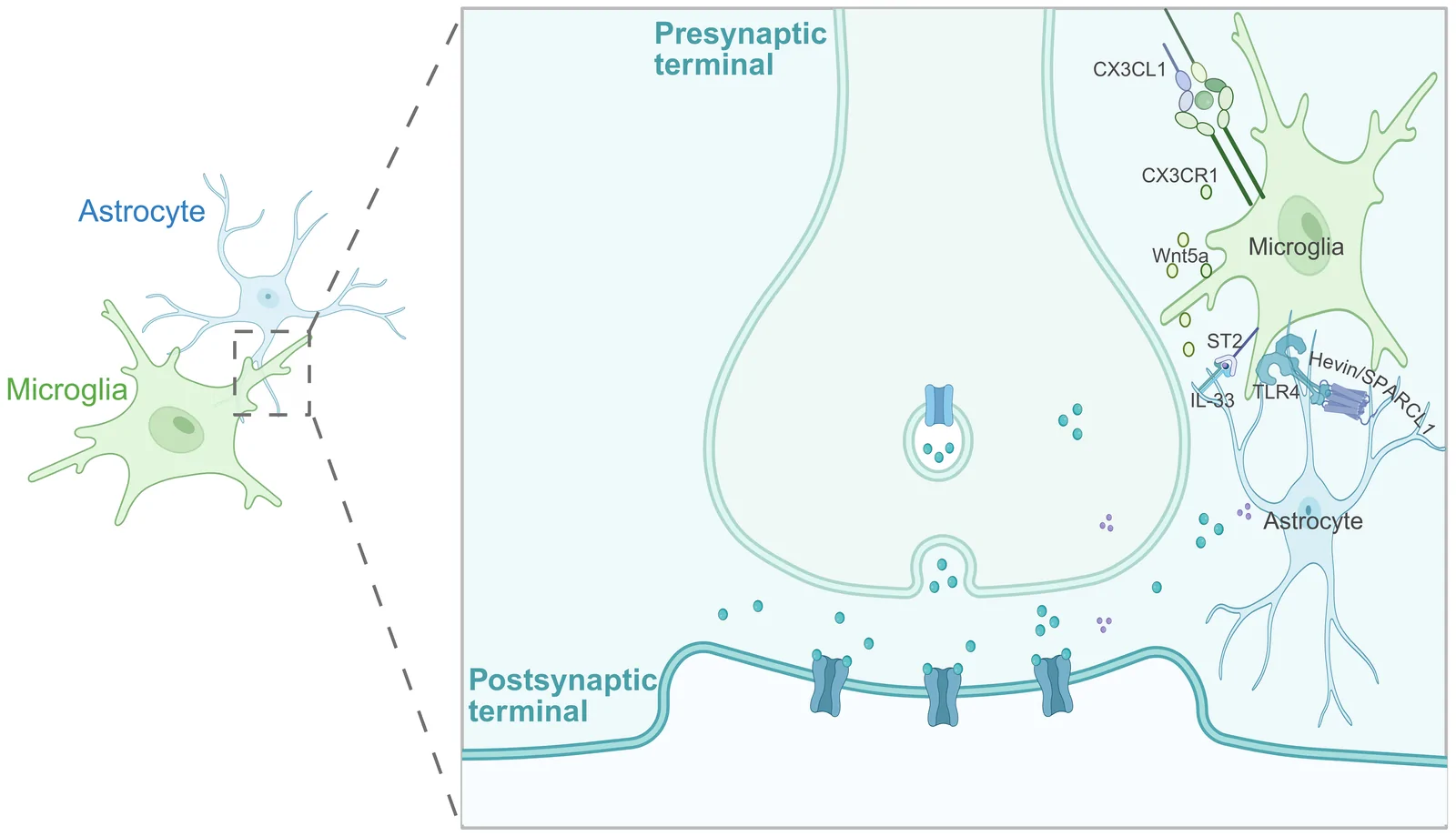
The role of glial crosstalk between astrocytes and microglia in the regulation of synaptic pruning. This schematic illustrates the reciprocal interactions between astrocytes and microglia in regulating synaptic homeostasis and remodeling. Neuronal CX3CL1/CX3CR1 signaling contributes to neuron–microglia communication and influences microglial responses. Microglia-derived Wnt signals modulate astrocytic regulation of synaptic structures, while astrocyte-derived factors, including Hevin/SPARCL1 and IL-33, regulate microglial activity through pathways such as TLR4 and ST2 signaling. These interactions influence microglial phagocytic function and synaptic remodeling, and their dysregulation may contribute to impaired synaptic plasticity associated with depression and other neuropsychiatric disorders. Created in BioRender. Yao, SX. (2026) https://BioRender.com/tc15t5c.

### 3.1 “Find Me” signals

In the process of microglia-mediated synaptic pruning, “Find Me” signals serve as critical molecular cues that guide microglial recruitment toward vulnerable or altered synaptic regions. In addition to classical chemotactic molecules, neurotransmitter-mediated signaling may also influence microglial recruitment and synaptic remodeling under pathological conditions. For example, GABA released from inhibitory neurons activates GABA-B receptors on microglia, inducing calcium signaling and promoting microglial accumulation near inhibitory synapses. This process may contribute to excessive elimination of inhibitory synapses under pathological conditions, thereby facilitating disease progression (11). Furthermore, the CX3CL1/CX3CR1 signaling axis represents a major neuron-microglia communication pathway involved in synaptic remodeling. The interaction between neuronal chemokine CX3CL1 and its receptor CX3CR1 on the microglial surface regulates dendritic spine dynamics and maintains synaptic homeostasis (12). The CX3CL1/CX3CR1 pathway contributes to microglial recruitment and functional regulation in response to neuronal activity changes. Recent studies have revealed that IL-15RA deficiency enhances microglial activation through this pathway, leading to excessive synaptic pruning and depressive-like phenotypes (13). Additionally, ATP and ADP released from neurons or glial cells serve as classical “Find Me” signals by activating P2Y12 receptors expressed on microglia. This purinergic signaling pathway promotes microglial process extension and chemotactic migration toward altered synaptic regions, thereby facilitating synaptic surveillance and remodeling. In the central nervous system, ATP functions not only as a neurotransmitter but also as an important neuromodulator, regulating neuronal activity and synaptic transmission through multiple pathways, particularly within inhibitory neurons and synapses (14). Beyond classical “Find Me” signals, non-classical modulators such as glutamate (15, 16), dopamine (17), and norepinephrine (18) may indirectly regulate microglial recruitment and pruning activity by modifying neuronal activity and microglial functional states. Collectively, “Find Me” signals establish the initial communication interface between neurons and microglia by directing microglial surveillance toward vulnerable synaptic regions. However, recruitment alone does not determine whether synapses are eliminated or preserved. The subsequent fate of synaptic structures is controlled by opposing “Eat Me” and “Don’t Eat Me” signals.

### 3.2 “Eat Me” signals

Following microglial recruitment by neuronal “Find Me” signals, “Eat Me” signals determine the recognition and engulfment of synaptic structures destined for elimination. Upon arrival at vulnerable synaptic regions, microglia recognize molecular tags on susceptible synapses and initiate phagocytic programs. Phosphatidylserine (PS) represents one of the important “Eat Me” signals involved in microglial recognition of vulnerable synaptic structures; under normal physiological conditions, PS is primarily restricted to the inner leaflet of the plasma membrane. However, when synapses undergo stress, damage, or developmental weakening, PS becomes externalized to the outer leaflet of the membrane. Exposed PS is recognized by TAM family receptors on microglia, such as MERTK and AXL, through bridging molecules including Gas6 and Pros1, thereby initiating phagocytic programs (19). Furthermore, TREM2 is a transmembrane receptor expressed on microglia that recognizes lipid-associated damage signals and regulates phagocytic activation. Through downstream signaling pathways, TREM2 promotes microglial responses to synaptic damage and facilitates the clearance of cellular debris and vulnerable synaptic structures (20). During early postnatal development, the canonical complement cascade serves as the primary mechanism for eliminating redundant or weaker synapses in the visual system. In this process, C1q initiates synaptic tagging, followed by activation of C3 and deposition of C3b/iC3b fragments on synaptic structures destined for removal. These complement-opsonized synapses are subsequently recognized by complement Receptor 3 (CR3, CD11b/CD18) on microglia, triggering engulfment (21). In parallel with complement-dependent synaptic tagging, TAM receptor signaling provides a complementary PS-dependent pathway for synaptic engulfment. TAM receptors, including MERTK and AXL, recognize PS-positive synaptic structures through Gas6 or Pros1, thereby promoting synaptic internalization (22). MERTK and TREM2 independently but complementarily regulate microglial synaptic phagocytosis, enabling microglia to integrate different “Eat Me” signals and modulate their engulfment behavior (23, 24). Collectively, “Eat Me” signals provide molecular tags that determine which synaptic structures are recognized and eliminated by microglia, thereby maintaining neural circuit refinement and homeostasis.

### 3.3 “Don’t Eat Me” signals

As a counter-regulatory mechanism opposing excessive “Eat Me” signaling, “Don’t Eat Me” signals play a critical protective role in microglia-mediated synaptic pruning. Their core function is to preserve functional synaptic structures by raising the threshold for microglial engulfment. Microglia express inhibitory receptors and homeostatic signaling pathways that sense these protective cues, thereby precisely regulating synaptic engulfment. Specifically, Lehrman et al. discovered that neuronal CD47 interacts with SIRPα on microglia, initiating inhibitory signaling that suppresses phagocytic activation and protects functional synapses from inappropriate elimination (25). At the signal transduction level, beyond CD47-SIRPα signaling, cell-surface sialic acids can engage Siglec receptors on microglia, generating inhibitory signals that restrain phagocytic activation. Siglec activation recruits inhibitory phosphatases such as SHP-1, which suppress phosphorylation of SYK and downstream substrates, thereby restraining phagocytic signaling (26). Furthermore, IL-34 functions as an important regulator of microglial homeostasis through CSF1R signaling. By maintaining a surveillance-oriented phenotype, IL-34 indirectly prevents excessive synaptic elimination (27). Collectively, these signaling pathways establish a balance between synaptic elimination and preservation, ensuring that microglial pruning remains within physiological limits and preventing neural circuit dysfunction caused by excessive synaptic loss.

### 3.4 Glial crosstalk in the regulation of synaptic pruning

The maintenance of synaptic homeostasis also depends on coordinated communication between microglia and other glial populations. Before microglia-mediated synaptic engulfment, astrocyte-synapse interactions undergo dynamic remodeling. This process involves neuronal-microglial CX3CL1/CX3CR1 communication and is additionally regulated by Wnt proteins released from microglia. Studies indicate that microglia can activity-dependently secrete Wnt signaling molecules, reducing astrocytic coverage of synaptic structures and creating a permissive microenvironment for microglial engulfment (28). Furthermore, Hevin/SPARCL1, a synaptogenic protein secreted by astrocytes, not only promotes the formation of thalamocortical synapses but also interacts with microglial TLR4 signaling through its C-terminal fragment. This signaling pathway enhances microglial phagocytic activity and promotes synaptic pruning, particularly during brain development (29). Additionally, astrocytes secrete IL-33, which binds to the ST2 receptor on the microglial surface. This not only promotes microglial phagocytic function (“Eat Me”) but also directly drives synaptic pruning, particularly in the developing brain (30).

## 4 Aberrant microglial synaptic pruning and impaired synaptic plasticity in depression

Synaptic plasticity represents a fundamental property of the nervous system that enables adaptive changes in neuronal connectivity and function. It refers to the capacity of synapses, which connect and coordinate neuronal communication, to undergo enduring structural and functional alterations in response to fluctuations in neural activity. This property is regarded as the biological substrate for learning, memory, and environmental adaptation, representing a central concept in neuroscience (31, 32). Synaptic plasticity is generally classified into short-term and long-term forms. These processes involve the modulation of presynaptic neurotransmitter release, changes in postsynaptic receptor activity, and regulation by calcium-dependent signaling pathways (33, 34). In this process, frequently activated pathways are strengthened, enabling more efficient information transmission; conversely, connections that are rarely used may be gradually eliminated through synaptic pruning, achieving the refinement and optimization of neural networks. This principle of “use it or lose it” not only shapes the functional circuits of the brain but also profoundly influences cognition, emotion, and behavioral adaptation, highlighting the dynamic capacity of neural circuits for continuous adaptation (35).

However, in the pathological environment of depression, this delicate equilibrium is disrupted. Chronic stress and persistent inflammatory signaling activate microglial complement pathways, characterized by increased C1q/C3 deposition and enhanced CR3-dependent synaptic engulfment. This pathological over-pruning, characterized by the non-selective elimination of vulnerable or weakly active synapses in emotion-regulating brain regions such as the prefrontal cortex and hippocampus, disrupts excitatory-inhibitory (E/I) circuit balance. It triggers a significant reduction in dendritic spine density and the downregulation of synaptic plasticity-related proteins (e.g., PSD-95, GluR1) (36–38). Ultimately, the structural and functional impairment of synaptic plasticity, manifested as the inhibition of long-term potentiation (LTP) and reduced synaptic transmission efficiency, contributes to the cellular mechanisms underlying cognitive and emotional dysregulation in depression. Therapeutic strategies targeting aberrant microglial pruning, including modulation of complement signaling pathways and restoration of physiological phagocytic homeostasis, are emerging as potential antidepressant approaches to reverse synaptic plasticity deficits and remodel neural circuit connectivity.

### 4.1 BDNF signaling dysfunction and microglia-mediated synaptic vulnerability

Brain-Derived Neurotrophic Factor (BDNF) is a critical protein essential for neuronal survival, growth, differentiation, and synaptic plasticity. By activating TrkB receptors, BDNF regulates neurotransmitter release, dendritic spine formation and stabilization, and processes such as long-term potentiation (LTP), thereby maintaining normal neural circuit function (39). In the pathogenesis of depression, BDNF signaling imbalance plays a central role. A decline in BDNF levels may compromise synaptic maintenance and increase susceptibility to excessive or aberrant synaptic pruning, thereby disrupting neural circuit stability (40). Chronic stress inhibits BDNF expression, resulting in weakened synaptic connections in the hippocampus and prefrontal cortex, which impairs emotional regulation and cognitive function (41, 42). BDNF function is characterized by a unique bidirectional regulation. Pro-BDNF preferentially activates p75NTR signaling, which can promote synaptic weakening, remodeling, or apoptosis under specific conditions. Conversely, mature BDNF (mBDNF) binds to TrkB receptors, initiating a signal transduction cascade that promotes synaptic stability, neuronal survival, and synaptic plasticity, thereby strengthening synaptic connections and maintaining normal neural network function (43–45). Abnormalities in BDNF-TrkB signaling impair downstream pathways, including PI3K/Akt/mTOR and MAPK/ERK/CREB, which regulate synaptic protein synthesis and plasticity-associated gene expression, including PSD-95 and GluA1, thereby increasing synaptic vulnerability and impairing synaptic structure and function (46). Furthermore, impaired MAPK/ERK/CREB signaling reduces the expression of plasticity-related genes, contributing to altered dendritic spine morphology and synaptic dysfunction (47). In addition, there is a close interaction between BDNF signaling and microglia-mediated synaptic pruning. Excessive microglial pruning reduces functional synaptic connectivity and decreases activity-dependent BDNF expression, whereas impaired BDNF signaling further weakens synaptic resilience and increases susceptibility to pathological synaptic elimination, thereby contributing to synaptic dysfunction in depression. In conclusion, BDNF signaling dysregulation and microglial pruning abnormalities form a pathological feedback loop ultimately leading to synaptic loss and circuit imbalance in the prefrontal cortex and hippocampus, constituting a key neuropathological mechanism of depression, as shown in **Figure 3**.

**Figure 3:**
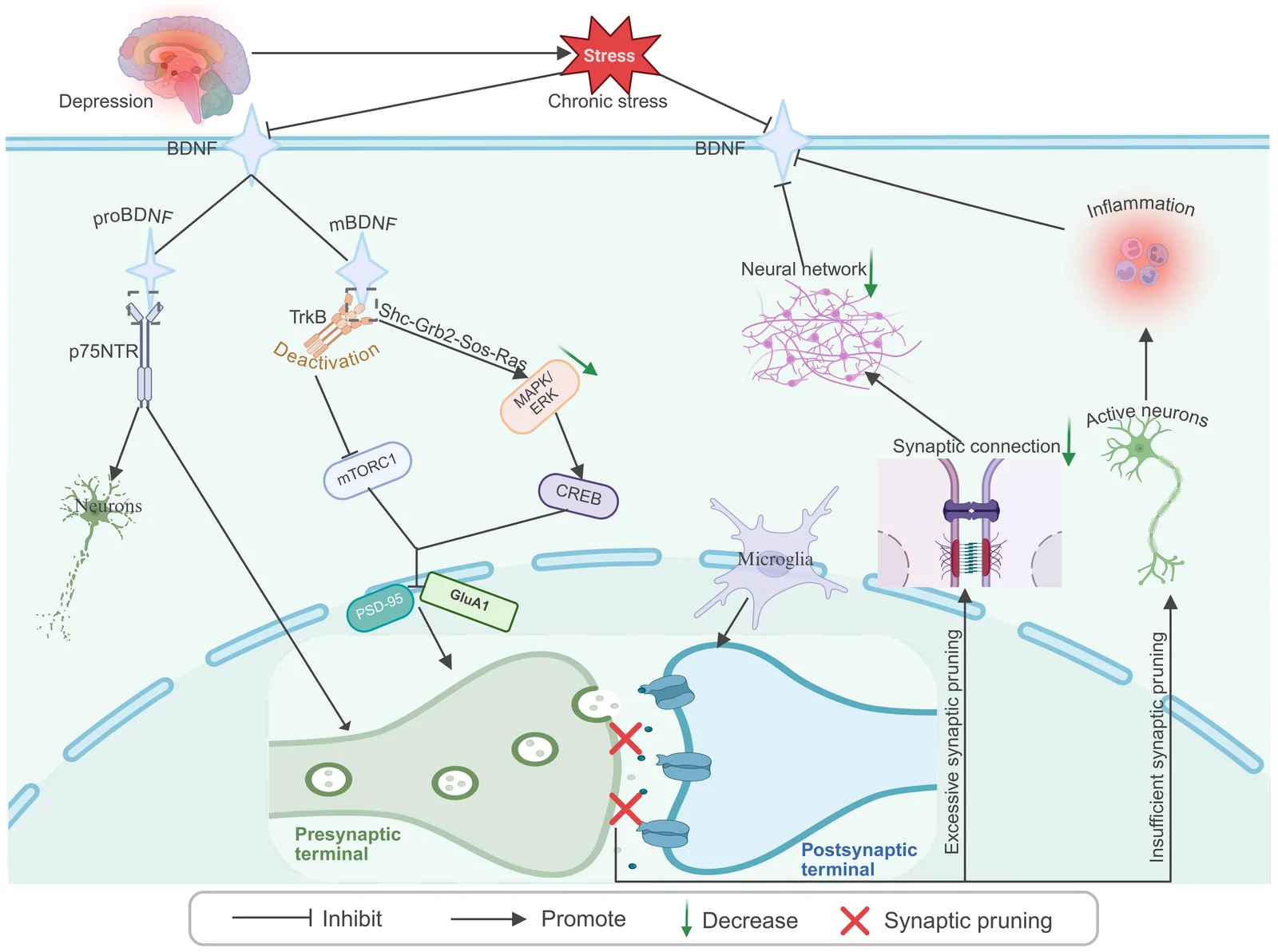
BDNF signaling dysfunction and microglia-mediated synaptic vulnerability. This schematic illustrates the interaction between BDNF signaling imbalance and microglia-mediated synaptic remodeling. Reduced BDNF expression and disrupted mBDNF/TrkB and proBDNF/p75NTR signaling impair downstream pathways, including PI3K/Akt/mTOR and MAPK/ERK/CREB, leading to reduced synaptic protein expression and impaired synaptic plasticity. Meanwhile, excessive microglial pruning may further decrease synaptic connectivity and may aggravate BDNF deficiency, forming a pathological feedback loop that contributes to synaptic dysfunction and depression. Created in BioRender. Yao, SX. (2026) https://BioRender.com/rkecxfk.

### 4.2 Neuroinflammatory activation drives microglial complement-dependent synaptic pruning

In addition to the deficiency of neurotrophic factors, immune system overactivation represents another critical force driving the imbalance in synaptic pruning. Studies indicate that levels of pro-inflammatory cytokines (such as IL-1β, IL-6, and TNF-α) are significantly elevated in patients with depression (48). Elevated pro-inflammatory cytokines promote microglial activation and phenotypic transition toward a pro-inflammatory state, enhancing inflammatory mediator release and phagocytic activity (49). When neuroimmune homeostasis is disrupted, microglia transition into a reactive state characterized by enhanced inflammatory and phagocytic activity. Complement components such as C1q and C3 opsonize vulnerable synapses, facilitating their recognition and engulfment by microglia and promoting excessive synaptic pruning. This excessive pruning results in synaptic loss and impaired neuronal connectivity, severely affecting functions in brain regions such as the prefrontal cortex and hippocampus (12). Chronic stress increases the expression of gene sets related to immune-inflammatory signaling in the prefrontal cortex and elevates complement C3 protein levels in the medial prefrontal cortex. This results in an increased co-localization of complement C3 with synaptic structures within microglia. This promotes excessive engulfment of excitatory synapses by microglia, leading to synaptic dysfunction and reduced cortical functional connectivity in adulthood, thereby inducing depressive-like behaviors in mice (50). Additionally, inflammatory cytokines activate the palmitoylation cycle (regulated by DHHC7 and APT2) within microglia, promoting STAT3 phosphorylation and nuclear translocation. This further reinforces inflammatory signaling and microglial activation, forming a positive feedback loop that exacerbates synaptic pruning and neuroinflammation (51). Meanwhile, chronic inflammation increases blood-brain barrier (BBB) permeability, allowing peripheral immune cells (such as monocytes) to infiltrate the brain. These infiltrating immune cells interact with microglia and astrocytes to form a sustained inflammatory microenvironment, directly damaging neurons and synaptic structures (52, 53). This inflammatory state also inhibits the expression of BDNF. Reduced BDNF availability further compromises synaptic resilience, amplifying inflammation-induced synaptic dysfunction (54, 55). Collectively, neuroinflammatory signals converge on microglial dysfunction, promoting complement-dependent excessive synaptic engulfment and disruption of neurotrophic support, thereby constituting a core neuroimmune mechanism underlying synaptic impairment in depression, as shown in **Figure 4**.

**Figure 4:**
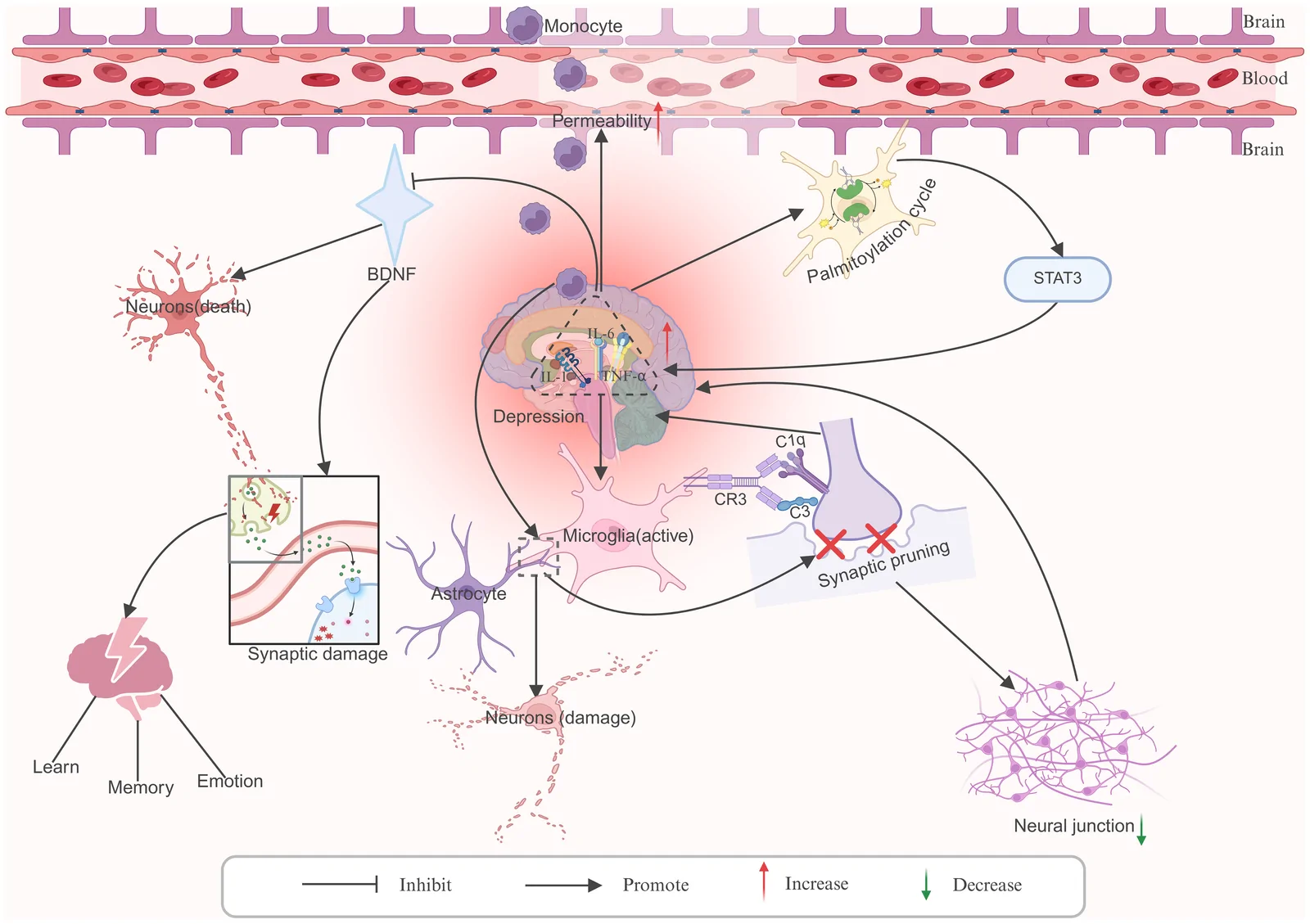
Neuroinflammatory activation drives microglial complement-dependent synaptic pruning. This schematic summarizes the role of neuroinflammation in promoting microglial activation and excessive synaptic elimination. Pro-inflammatory cytokines activate microglia, while complement factors C1q and C3 facilitate synaptic recognition and engulfment through microglial receptors, potentially leading to synaptic loss and impaired neural connectivity. Inflammatory signaling, including the palmitoylation-STAT3 pathway, further reinforces microglial activation. Meanwhile, BBB disruption, peripheral immune infiltration, and reduced BDNF availability contribute to a sustained inflammatory environment and increased synaptic vulnerability. Created in BioRender. Yao, SX. (2026) https://BioRender.com/d2pdlb1.

### 4.3 Neurotransmitter dysregulation promotes microglia-mediated synaptic pruning

Neurotransmitters are chemical messengers that transmit signals between neurons, playing a pivotal role in regulating mood, cognition, and behavior. When a neurotransmitter imbalance occurs, it not only affects signal transmission between neurons but also impairs synaptic plasticity. Microglia express various serotonin receptors on their surface (e.g., 5-HT1B, 5-HT2B). Under physiological conditions, serotonin signaling exerts inhibitory effects on microglial activation, maintaining microglia in a surveillant homeostatic state. In depression, reduced serotonergic signaling weakens this inhibitory regulation, rendering microglia more susceptible to adopting a pro-inflammatory phenotype (56–59). Concurrently, patients with depression often exhibit abnormalities in the glutamate system. Excessive glutamate not only directly damages neurons through excitotoxicity but also promotes microglial activation and inflammatory cytokine release (60, 61). Inflammatory signaling promotes complement deposition (e.g., C1q and C3) on vulnerable synapses, facilitating their recognition and engulfment by microglia through complement receptors such as CR3. Activated microglia subsequently engulf these complement-opsonized synapses, resulting in pathological synaptic over-pruning (62, 63). As a result, excessive pruning may eliminate not only damaged synapses but also functionally intact synaptic connections. The loss of synaptic connectivity and structural integrity leads to attenuation of long-term potentiation (LTP), thereby impairing activity-dependent synaptic strengthening (64–66). In summary, neurotransmitter imbalance, particularly reduced serotonergic signaling and glutamatergic dysregulation, promotes aberrant microglial activation and complement-dependent synaptic pruning, ultimately contributing to synaptic loss, impaired synaptic plasticity, and the pathophysiology of depression, as shown in **Figure 5**.

**Figure 5:**
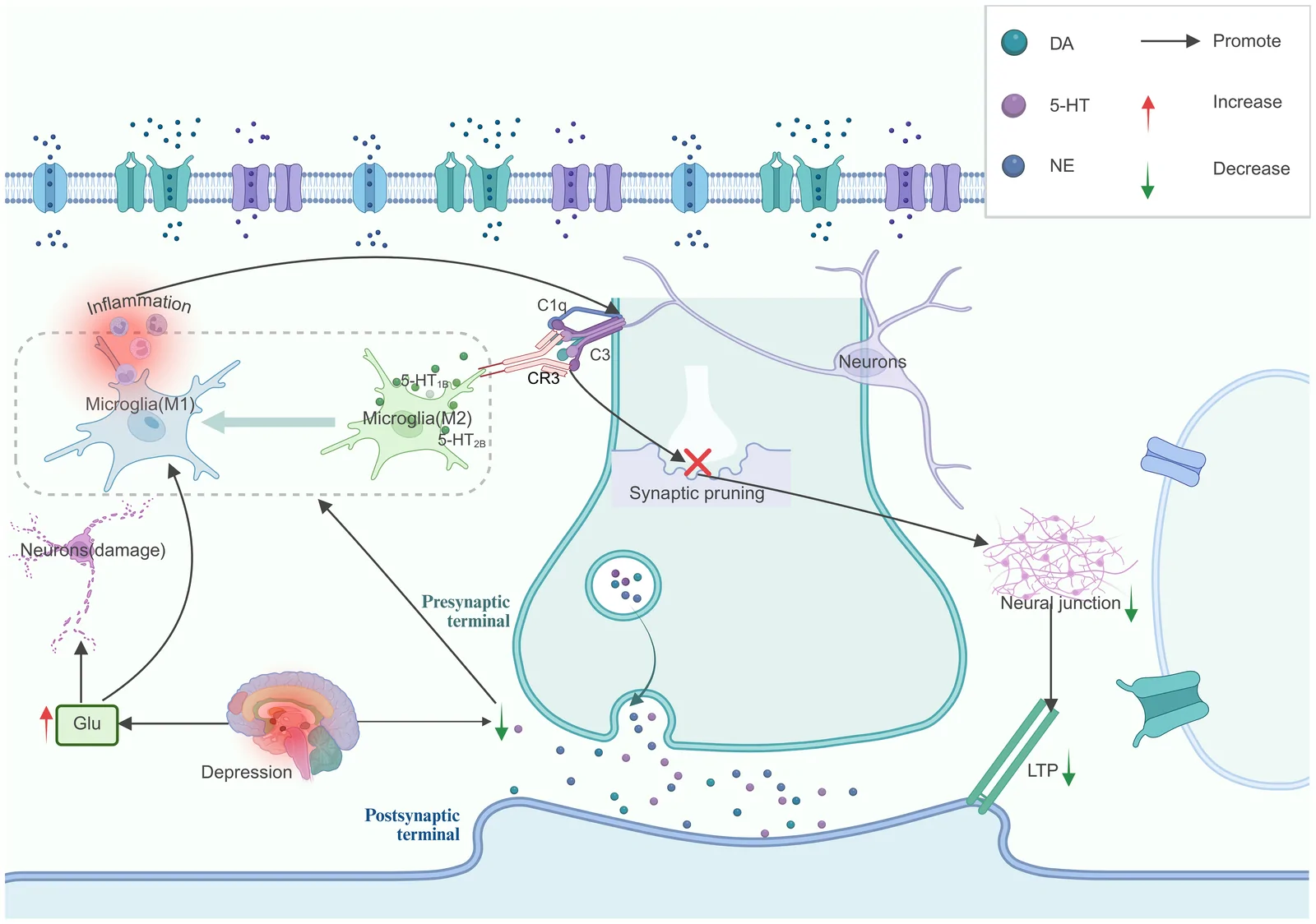
Neurotransmitter dysregulation promotes microglia-mediated synaptic pruning. This schematic summarizes the role of neurotransmitter imbalance in regulating microglial activation and synaptic elimination. Reduced serotonin signaling and excessive glutamate activity promote microglial pro-inflammatory responses, leading to complement-dependent synaptic engulfment through C1q/C3 and CR3 pathways. Excessive synaptic pruning disrupts neuronal connectivity and impairs synaptic plasticity, including LTP, contributing to depression-related circuit dysfunction. Created in BioRender. Yao, SX. (2026) https://BioRender.com/rh8vbbu.

### 4.4 Chronic stress-induced HPA axis dysregulation and synaptic pruning

Chronic stress-induced depression involves neuroendocrine dysregulation, inflammatory activation, and synaptic structural remodeling. Persistent HPA axis dysregulation promotes neuroinflammation and aberrant microglial activation, thereby facilitating pathological synaptic pruning. This process initiates with the overactivation of the hypothalamic-pituitary-adrenal (HPA) axis, leading to sustained elevation of glucocorticoids (such as cortisol). These hormones subsequently affect gene expression and cellular signaling pathways via the glucocorticoid receptor (GR) and its chaperone protein FKBP5, resulting in dendritic atrophy, reduced synaptic number, and decreased synaptic plasticity, thereby disrupting brain homeostasis (67). Under chronic stress, high concentrations of cortisol induce the aberrant overexpression of FKBP5, which binds tightly to the GR complex. This reduces GR sensitivity to glucocorticoids, leading to glucocorticoid resistance (68, 69). This resistance not only disrupts the negative feedback regulation of the HPA axis, perpetuating the high cortisol state, but also impairs GR-mediated transcription of neuroprotective genes, including BDNF, and induces neuroinflammation, thereby creating a permissive environment for microglia-mediated synaptic elimination. Consequently, chronic stress induces dendritic remodeling and synaptic loss in brain regions such as the prefrontal cortex and hippocampus, transforming adaptive acute stress responses into persistent structural impairment (70). Recent evidence has demonstrated that while acute stress activates autophagy as a protective cellular mechanism, chronic stress suppresses this process in lateral habenula (LHb) neurons. Impaired autophagic clearance causes accumulation of postsynaptic glutamate receptors, resulting in pathological LHb hyperactivity and inhibition of reward circuits, thereby contributing to depressive-like behaviors (71). Chronic stress also directly enhances microglia-mediated synaptic pruning. Recent evidence demonstrated that chronic stress downregulates Dkk3 expression in hippocampal CA1 neurons, activating the Wnt-CX3CL1/CX3CR1 signaling pathway and enhancing microglial engulfment of excitatory synapses, thereby linking chronic stress to pathological synaptic elimination (36). Collectively, chronic stress promotes HPA axis dysregulation, neuroinflammatory activation, and microglia-mediated synaptic pruning through glucocorticoid signaling abnormalities and stress-responsive molecular pathways. These processes ultimately contribute to synaptic loss and impaired neural circuit function in depression, as shown in **Figure 6**.

**Figure 6:**
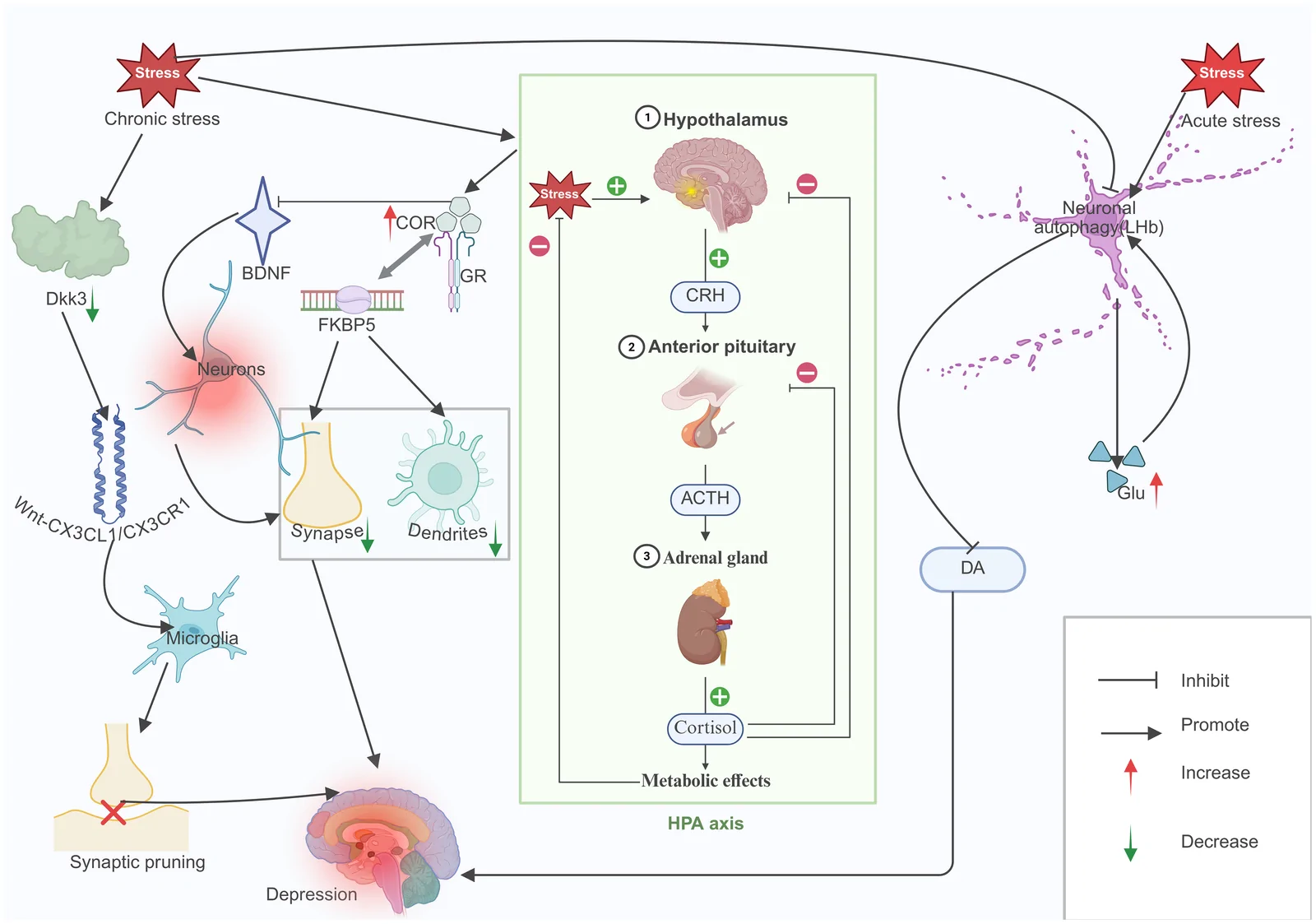
Chronic stress-induced HPA axis dysregulation and synaptic pruning. This schematic summarizes the effects of chronic stress on HPA axis activation, glucocorticoid signaling, and synaptic remodeling. Sustained cortisol elevation induces FKBP5-mediated GR dysfunction, reduces BDNF expression, and impairs synaptic stability. Chronic stress also enhances microglial synaptic engulfment through Dkk3/Wnt-CX3CL1/CX3CR1 signaling and contributes to neuroinflammation. Impaired autophagy and glutamatergic dysfunction further disrupt neural circuits, ultimately promoting depression-related synaptic deficits. Created in BioRender. Yao, SX. (2026) https://BioRender.com/yt46w97.

### 4.5 Gut microbiota dysbiosis promotes microglia-mediated synaptic pruning

Gut microbiota dysbiosis has emerged as an important contributor to depression through the microbiota-gut-brain axis. Increasing evidence suggests that microbial dysbiosis disrupts synaptic plasticity by altering microbial metabolites, neuroimmune signaling, and microglia-mediated synaptic pruning (72). One important mechanism underlying these effects is the disruption of synaptic plasticity. In depression models, researchers have observed a reduction in certain gut bacterial species, including Bifidobacterium longum and Roseburia intestinalis, as well as a decrease in the neurotransmitter metabolite homovanillic acid (HVA). HVA enters the brain by crossing the blood-brain barrier and accumulates predominantly in the hippocampus. It inhibits excessive autophagy by regulating LC3 and p62, preserves SYN1 expression, and maintains synaptic integrity and neuronal function, thereby reducing susceptibility to pathological synaptic pruning (73). Studies have found that gut microbiota specifically impair the structural connectivity and functional transmission of the VTA-mPFC dopaminergic pathway via the gut-brain axis, leading to reduced synaptic plasticity in the mPFC and thereby inducing depressive-like behaviors (74). Mechanistically, gut microbiota disruption induces IPA/AHR/NF-κB signaling dysregulation, promoting microglial activation and excessive synaptic pruning in the hippocampus. In parallel, microbial dysbiosis also activates complement C3 signaling, further enhancing microglia-mediated synaptic engulfment (75). Another study revealed a novel gut microbiota-brain pathway: the depletion of specific microbial taxa (f_Lachnospiraceae, f_Oscillospiraceae, and f_Erysipelotrichaceae) leads to decreased inosine levels, attenuating A2aR-PKA-CREB-HDAC3 signaling, increasing H3K9ac, and promoting EZH2 transcription. Subsequently, EZH2 induces H3K27me3 reprogramming, suppressing genes critical for serotonergic synaptic function, ultimately resulting in depressive behavior (76). Therefore, gut microbiota imbalance can remotely regulate synaptic plasticity in the central nervous system through multiple mechanisms, including the reduction of key metabolites (such as HVA, IPA, and inosine), aberrant activation of the complement system, and microglia-mediated excessive synaptic pruning, thereby contributing to synaptic dysfunction and the pathophysiology of depression. These findings provide a theoretical basis for developing microbiota-targeted therapeutic strategies aimed at restoring microglial homeostasis and synaptic plasticity in depression, as shown in **Figure 7**.

**Figure 7:**
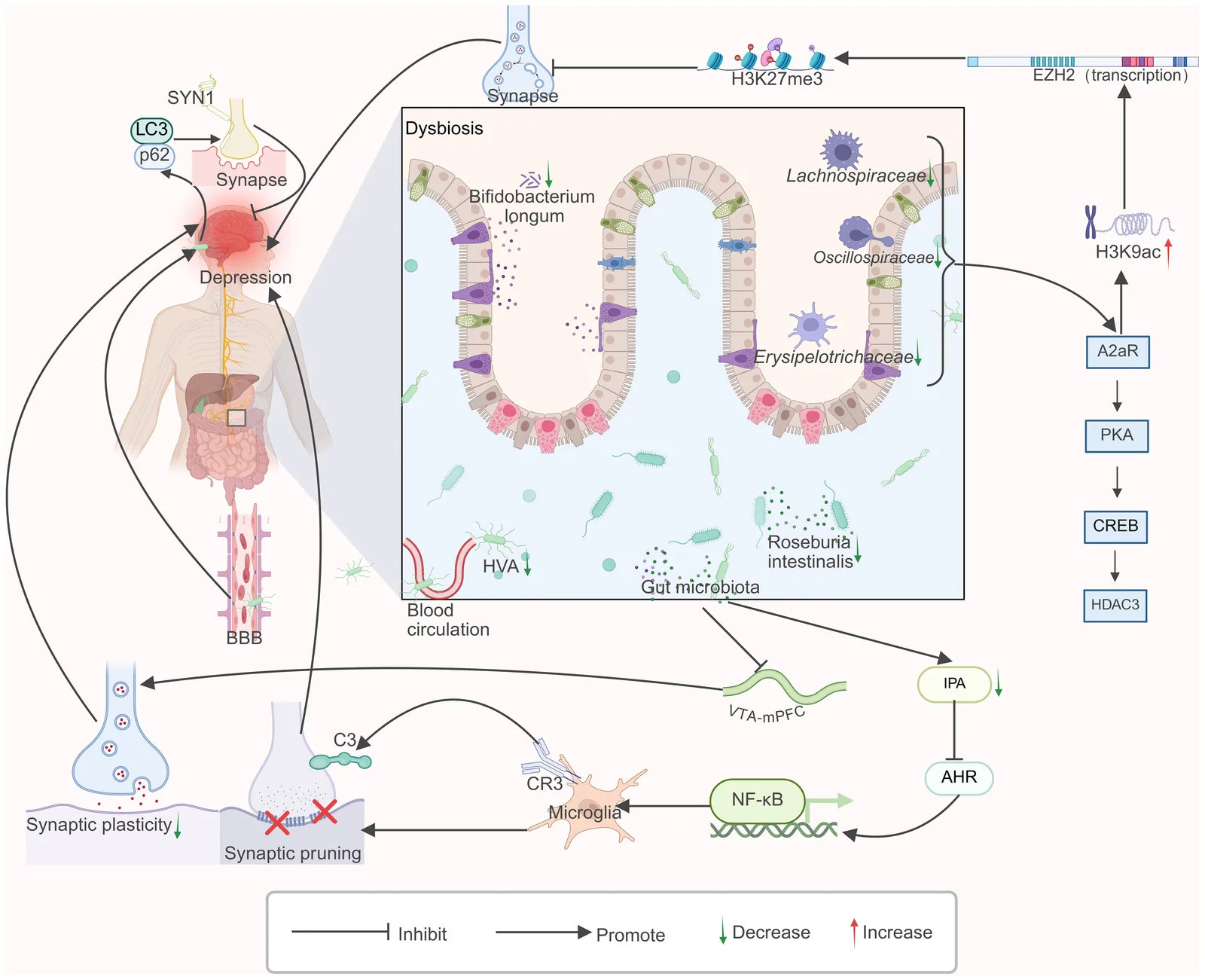
Gut microbiota dysbiosis promotes microglia-mediated synaptic pruning. This schematic summarizes the mechanisms by which gut microbiota imbalance regulates synaptic plasticity and microglial synaptic pruning through the microbiota-gut-brain axis. Altered microbial composition and reduced metabolites, including HVA, IPA, and inosine, impair synaptic maintenance, immune regulation, and neuronal signaling. In related neurodevelopmental models, microbial dysbiosis has been shown to activate through IPA/AHR/NF-κB and complement C3 pathways, facilitating excessive synaptic engulfment. Meanwhile, microbiota-derived signals influence epigenetic regulation of synaptic function through A2aR-PKA-CREB-HDAC3 and EZH2-related pathways, ultimately contributing to synaptic dysfunction in depression.Created in BioRender. Yao, SX. (2026) https://BioRender.com/4e2tjuz.

### 4.6 Mitochondrial dysfunction promotes microglia-mediated synaptic pruning

Mitochondria serve not only as the primary source of cellular energy but also as key regulators of microglial phenotypic transition and phagocytic function (77, 78). Under physiological conditions, resting microglia rely on efficient oxidative phosphorylation (OXPHOS) for energy supply. This metabolic mode supports their delicate process motility, enabling them to gently and precisely identify and prune redundant synapses, thereby maintaining neural circuit homeostasis and plasticity. However, mitochondrial dysfunction is frequently accompanied by metabolic reprogramming from oxidative phosphorylation (OXPHOS) toward glycolysis, a metabolic shift closely associated with pro-inflammatory microglial activation (79). Recent studies demonstrated that the mitochondrial protein CISD1 plays a critical role in the pathogenesis of depression. Under chronic stress, CISD1 expression is significantly upregulated in microglia, promoting a metabolic shift toward glycolysis by altering the NAD+/NADH ratio. This metabolic reprogramming directly triggers microglial inflammatory activation, which in turn drives pathological synaptic pruning, resulting in depressive-like behaviors (37). Meanwhile, PGC-1α, a key regulator of mitochondrial biogenesis, is essential for maintaining microglial metabolic homeostasis and synaptic integrity. Studies indicate that PGC-1α deficiency impairs mitochondrial function and cellular bioenergetics in microglia. This bioenergetic crisis forces microglia to abandon physiological homeostatic surveillance and switch to a hyperphagocytic phenotype. This metabolic defect-driven excessive synaptic pruning ultimately results in synaptic loss, impaired synaptic plasticity, and cognitive dysfunction (80). In summary, mitochondrial dysfunction promotes metabolic reprogramming, inflammatory microglial activation, and excessive synaptic pruning, ultimately contributing to synaptic loss, impaired synaptic plasticity, and the pathophysiology of depression, as shown in **Figure 8**.

**Figure 8:**
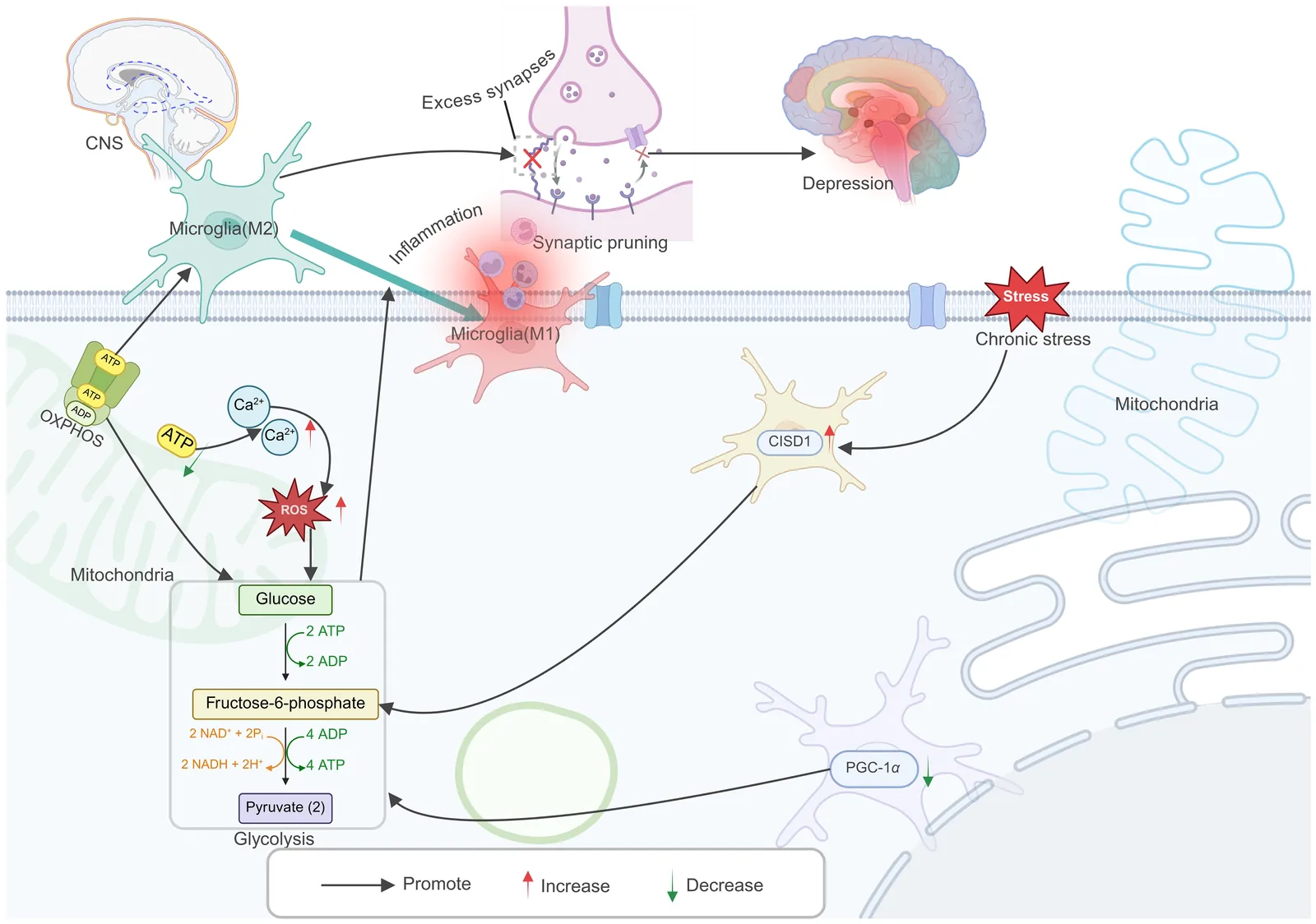
Mitochondrial dysfunction promotes microglia-mediated synaptic pruning. This schematic summarizes the role of mitochondrial dysfunction in regulating microglial metabolic state and synaptic remodeling. Impaired mitochondrial function induces a shift from oxidative phosphorylation toward glycolysis, accompanied by increased oxidative stress and inflammatory microglial activation. CISD1 upregulation and PGC-1α deficiency further disrupt metabolic homeostasis, promoting excessive microglial phagocytic activity and synaptic elimination. These metabolic abnormalities contribute to synaptic loss, impaired plasticity, and depression-related neural circuit dysfunction. Created in BioRender. Yao, SX. (2026) https://BioRender.com/6df89eg.

### 4.7 Epigenetic alterations regulate microglia-mediated synaptic pruning

Epigenetic alterations have emerged as important regulators of microglial function and synaptic plasticity. Increasing evidence indicates that epigenetic mechanisms may modulate microglial activation and complement-dependent synaptic pruning, potentially contributing to synaptic alterations associated with depression (81). Epigenetic mechanisms regulate microglial activation and synaptic pruning through both cell-intrinsic and neuron-derived pathways. However, some mechanistic insights are derived from developmental or other neurological disease models rather than depression-specific studies. Under normal physiological conditions, SETDB1 maintains endogenous retroviruses (ERVs) in a state of transcriptional silencing by catalyzing H3K9me3 modification. However, studies in autism-related and maternal immune activation models have shown that SETDB1 deficiency or maternal immune activation can release this epigenetic repression, allowing ERVs to be reactivated. The activated ERVs accumulate as RNA-DNA hybrids in the neuronal cytoplasm. These hybrids drive the aberrant overexpression of the complement molecule C4b in neurons. Acting as a complement opsonin, C4b facilitates microglial recognition of vulnerable synapses, promoting excessive synaptic engulfment in the prefrontal cortex. This ultimately leads to decreased synaptic density and neural circuit dysfunction in these experimental models (82). Although these findings were not obtained directly from depression models, they provide valuable mechanistic evidence for understanding how epigenetic dysregulation may influence complement-dependent synaptic remodeling. Within microglia, the lipid phosphatase SHIP1 functions as a negative regulator of complement-mediated synaptic pruning. Studies using developmental models have shown that deletion of SHIP1 during early development enhances complement signaling, triggering excessive synaptic loss and resulting in cognitive deficits in adulthood (83). In summary, existing evidence suggests that epigenetic dysregulation may influence complement-dependent microglial synaptic pruning through both neuronal and microglial mechanisms, potentially contributing to synaptic vulnerability and impaired plasticity in depression. However, direct evidence from depression models remains limited, as shown in **Figure 9**.

**Figure 9:**
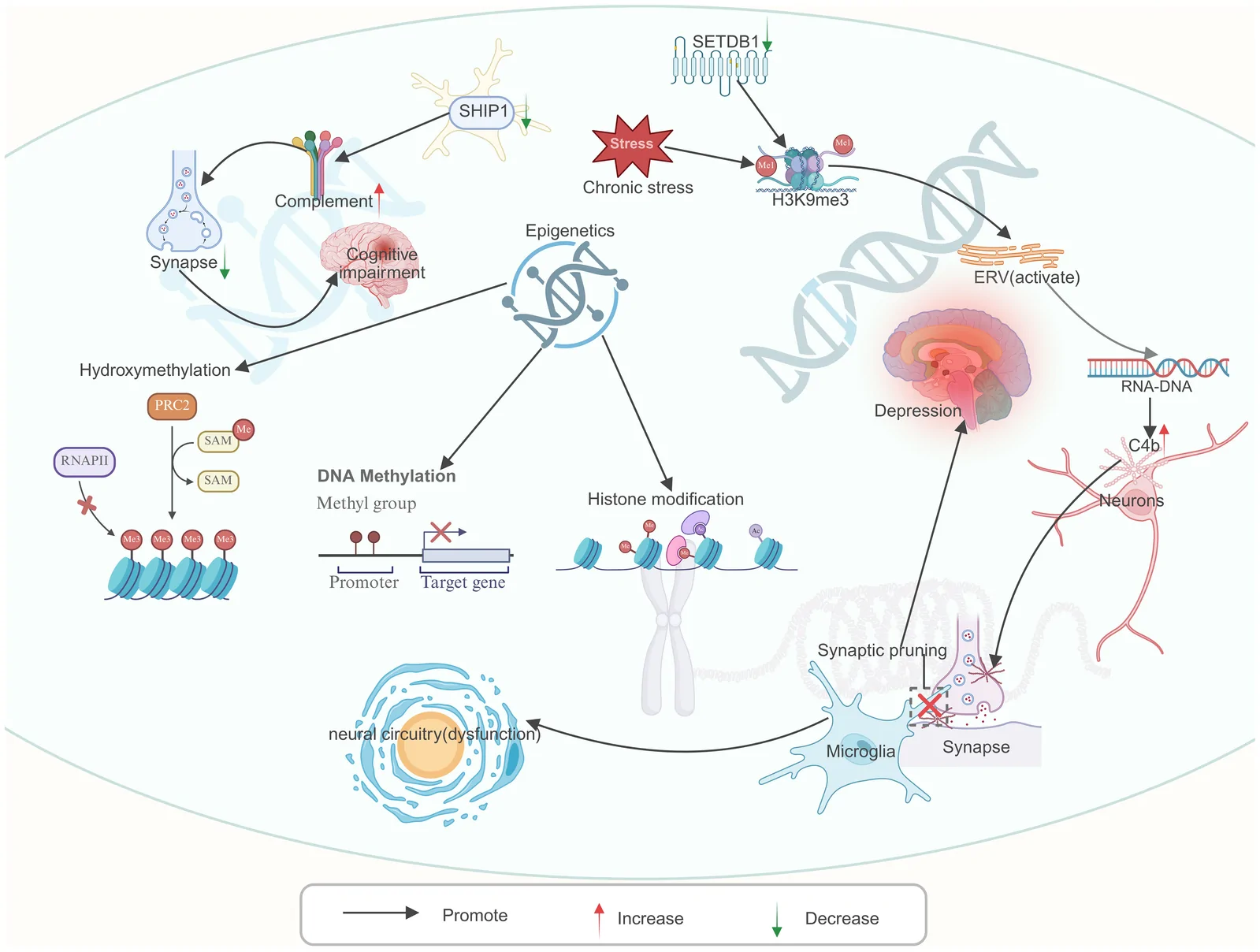
Potential epigenetic regulation of complement-dependent microglial synaptic pruning. This schematic summarizes the role of epigenetic mechanisms in regulating complement-dependent microglial synaptic pruning. Neuronal SETDB1 deficiency induces ERV activation, RNA-DNA hybrid formation, and C4b upregulation, promoting microglial recognition of synapses. Meanwhile, microglial SHIP1 deficiency enhances complement signaling and synaptic engulfment. These converging pathways provide mechanistic insights into how epigenetic dysregulation may influence complement-dependent synaptic remodeling, although their direct relevance to depression remains to be established. Created in BioRender. Yao, SX. (2026) https://BioRender.com/lhid8kt.

## 5 Traditional Chinese medicine-mediated modulation of pruning-associated pathways at the microglia-synapse interface

In recent years, increasing attention has been directed toward the regulation of synaptic plasticity in depression, encompassing multiple intervention categories, including TCM formulas, single herbs, and bioactive compounds derived from traditional medicinal sources. Based on the molecular mechanisms underlying microglia-mediated synaptic pruning described above, TCM interventions may potentially modulate the microglia-synapse interface through multiple biological processes, including regulation of neurotrophic signaling, modulation of neuroinflammatory responses, regulation of glutamatergic function, and improvement of mitochondrial homeostasis. However, most current studies evaluate the antidepressant effects of TCM interventions primarily through changes in synaptic plasticity-related markers, inflammatory mediators, or neuronal structural alterations, rather than direct assessment of microglial synaptic engulfment. Therefore, evidence supporting the regulation of microglia-mediated synaptic pruning by TCM remains limited, with most available findings representing indirect or associative evidence. This section summarizes current evidence from three intervention categories: TCM formulas, single herbs, and bioactive compounds derived from traditional medicinal sources, with a focus on their potential modulation of pruning-associated pathways at the microglia-synapse interface. as shown in **Figure 10**.

**Figure 10:**
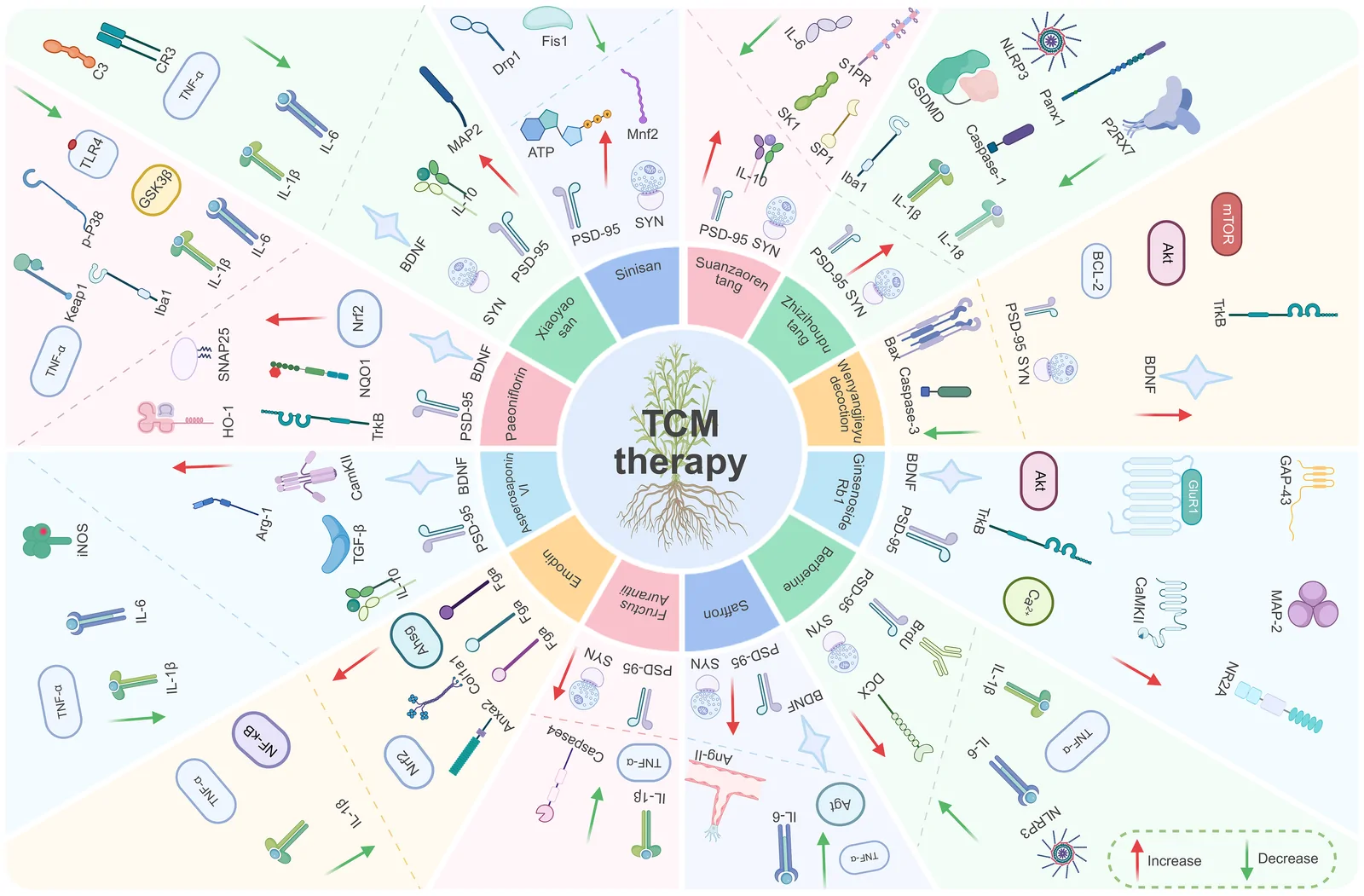
Traditional Chinese medicine modulates the microglia-synapse interface through pruning-associated pathways in depression. This schematic summarizes representative TCM formulas and bioactive compounds reported to improve depression-related synaptic dysfunction through regulation of neuroinflammation, neurotrophic signaling, neurotransmission, mitochondrial homeostasis, and synaptic plasticity pathways. These interventions may influence microglial activation states and pruning-associated pathways, thereby potentially contributing to restoration of neural circuit function. Current evidence is mainly derived from preclinical studies, and direct evidence demonstrating TCM-mediated regulation of microglial synaptic engulfment remains limited. Created in BioRender. Yao, SX. (2026) https://BioRender.com/i60bxgt.

### 5.1 Traditional Chinese medicine formulas

TCM formulas represent a major component of traditional Chinese medicine practice. Their characteristic multi-component and multi-target properties may provide a potential framework for addressing the complex pathological networks involved in depression. Existing studies suggest that these formulas may influence the microglia-synapse interface through multiple mechanisms, including enhancement of neurotrophic signaling, modulation of neuroinflammatory responses, and regulation of synaptic structural proteins. However, most reported effects on synaptic plasticity represent indirect evidence, as alterations in microglial synaptic engulfment have rarely been directly examined. The following sections summarize the available evidence according to their potential relevance to pruning-associated mechanisms at the microglia-synapse interface, as summarized in **Table 1**.

**Table 1:** Summary of traditional Chinese medicine formulas associated with pruning-related pathways and synaptic plasticity alterations in depression models.

Name	Main composition	Animal/cell model	Dose/duration of administration	Pruning-associated pathway	Effects and mechanisms	Evidence level	References
Xiaoyaosan	*Radix Bupleuri* (Chaihu)*, Radix Angelicae sinensis* (Danggui)*, Radix Paeoniae alba* (Baishao)*, Rhizome Atractylodis macrocephala* (ChaoBaizhu), *Poria cocos* (Fuling), *Herba Menthae haplocalycis* (Bohe), *Rhizome Zingiberis officinale* (Shengjiang), and *Radix Glycyrrhizae preparata* (ZhiGancao)	CUMS rat model	i.g. 2.224 g·kg^-1^ for 4 weeks	BDNF/TrkB-associated synaptic resilience (indirect relevance to pruning susceptibility)	MAP-2;PSD-95; synaptophysin;BDNF;TrkB; p-TrkB↑	Indirect evidence	(84)
Xiaoyaosan	*Bupleurum chinense* DC*.*, *Paeonia lactiflora* Pall., *Angelica sinensis* (Oliv.) Diels, *Atractylodes lancea* (Thunb.) DC., *Wolfiporia extensa* (Peck) Ginns. *(syn. Poria cocos* (Schwein.) F.A. Wolf), *Glycyrrhiza glabra* L., *Mentha canadensis* L., and *Zingiber officinale* Roscoe	CUMS mice model	p.o. 0.658 g·kg^-1^ for 4 weeks	Complement C3/CR3-associated synaptic remodeling; microglial inflammatory regulation	IL-1β;IL-6;TNF-α; C3/CR3↓ IL-10; SYN;PSD-95↑	Associative evidence	(85)
Mume Pill	*Armeniaca mume Siebold; Coptidis Rhizoma; Phellodendron chinense Schneid.; Panax ginseng C. A. Mey.; Angelica sinensis (Oliv.) Diels; Cinnamomi Ramulus; Asari Radix et Rhizoma; Rhizoma Zingiberis; Aconiti Lateralis Radix Praeparata; Zanthoxylum bungeanum Maxim.*	CUMS rat model	i.g. 2.27 g·kg^-1^ for 8 weeks	BDNF/TrkB-associated synaptic maintenance and neuronal plasticity pathways	SYP;PSD-95;p-CaMK2;p-CREB; BDNF; p-TrkB; p-PLCγ1; IP3R↑	Indirect evidence	(86)
Wenxiao San	Pinelliae Rhizoma Praeparatum Cum Alumine; Caulis Bambusae in Taeniam; Chaihu-Baishao Immaturus; Citri Reticulatae Pericarpium; Angelica sinensis; Radix Paeoniae Alba Praeparata; Poria cocos (Schw.) Wolf;	CUMS rat model	i.g. 30.15, 60.3, 120.6 g·kg ^-1^ for 4 weeks	BDNF/TrkB-ERK/CREB signaling associated with synaptic resilience	BDNF; p-TrkB/TrkB; p-ERK/ERK; p-CREB/CREB↑	Indirect evidence	(87)
Wenyangjieyu Decoction	*Epimedium brevicornu Maxim.;Curculigo orchioides Gaertn.;Morinda officinalis How; Bupleurum chinense DC;Angelica sinensis (Oliv.) Diels; Anemarrhena asphodeloides Bge.; Phellodendron amurense Rupr.; Atractylodes macrocephala Koidz.;Paeonia lactiflora Pall.; Poria cocos (Schw.) Wolf; Mentha haplocalyx Briq.;Glycyrrhiza uralensis Fisch.;Zingiber officinale Rosc.*	MS+RS mice model	p.o. 66.84 g·kg^-1^ for 90 days	BDNF/TrkB-Akt/mTOR signaling associated with synaptic maintenance and neuronal resilience	PSD-95;Syn; Bcl-2; BDNF;p-TrkB/TrkB;p-Akt/Akt;p-mTOR/mTOR↑ Bax;Caspase-3↓	Indirect evidence	(88)
Baihe Dihuang Tang	Lilium brownii var. viridulum (fresh bulbs of Lilium brownii) and Rehmannia glutinosa (fresh roots of Rehmannia glutinosa)	chronic restraint stress (CRS) mouse model	i.g. 0.02 g·mL^-1^ for 14 days	BDNF/TrkB-associated synaptic resilience and neuronal stress regulation	PSD-95;SYN1; BDNF; p-TrkB↑ MAOA↓	Indirect evidence	(89)
Xiaoyao Kangai Jieyu Recipe	*Radix Bupleuri;Radix Angelicae Sinensis;Radix Paeoniae Alba;Spica Prunellae;Rhizoma Paridis;Rhizoma Curcumae Longae;Herba Hyperici Perforati;Radix et Rhizoma Ginseng*	BCRD mice model	p.o. 26.5 g·kg^-1^ for 21 days	BDNF/CREB-PI3K/Akt signaling associated with synaptic resilience	SYN;PSD-95;BDNF;CREB; PI3K;p-Akt↑	Indirect evidence	(90)
Zhizi Houpotang	Magnolia officinalis, Gardenia jasminoides, Citrus aurantium	CUMS mouse model	i.g. 6 g·kg^-1^ for 4 weeks	Microglial inflammatory activation pathways (Iba1/P2RX7/NLRP3 inflammasome) associated with synaptic remodeling	PSD-95;Synapsin1↑ Iba1;P2RX7;IL-1β; IL-18; Panx1; NLRP3;ASC;Caspase-1;GSDMD↓	Associative evidence	(91)
Compound Chaijin Jieyu Formula	*Radix Bupleuri; Radix Curcumae; Herba Hyperici Perforati; Rhizoma Curcumae Longae; Folium Perillae; Radix Paeoniae Alba; Rhizoma Anemarrhenae; Poria; Radix Polygalae; Rhizoma Acori Tatarinowii; Radix et Rhizoma Ginseng*	CUMS rat model	i.g. 2.835, 5.67, 11.34 g·mL^-1^ for 42 days	TLR4/NLRP3 inflammasome-associated microglial inflammatory activation and synaptic remodeling	PSD-95;SYN↑ ROS;IL-1β;TLR4;NLRP3↓	Associative evidence	(92)
Xingnao Jieyu Decoction	Acorus tatarinowii, Polygala, Salvia, Bupleurum, Albizia Julibrissin, turmeric, and Morinda officinalis.	PSD rat model	i.g. 10.5, 21 g·kg^-1^ for 21 days	Neuroimmune regulation and BDNF-associated synaptic resilience pathways	IL-4;IL-10;BDNF; PSD-95;SYN↑ IL-6;TNF-α;IL-1*β*↓	Indirect evidence	(93)
Suanzaoren Tang	*Semen Ziziphi Spinosae; Poria; Rhizoma Anemarrhenae; Rhizoma Chuanxiong; Radix Glycyrrhizae*	social isolation combined with CUMS rat model	i.g. 2.5, 5, 10 g·kg^-1^ for 4 weeks	SP1/SK1/S1PR1-associated neuroimmune regulation and synaptic remodeling pathways	SYN1;PSD-95;IL-10↑ SP1;SK1;S1PR;IL-6↓	Associative evidence	(94)
Jiaweibuyanghuanwu Decoction	*Radix Astragali; Angelicae Sinensis Radix; Paeoniae Radix Rubra; Pheretima; Chuanxiong Rhizoma; Carthami Flos; Persicae Semen; Bupleuri Radix; Curcumae Radix*	MCAO rat model and social isolation combined with CUMS rat model	i.g. 6.7, 13.4, 26.8 g·kg^-1^ for 3 weeks	Microglial inflammatory phenotype regulation associated with synaptic remodeling	iNOS; IL-6;TNF-α;IL-1*β*↓ Arg-1;CD206; IL-10;TGF-*β*; SYN1;PSD-95↑	Associative evidence	(95)
Wenyang Jieyu Prescription	*Curculiginis Rhizoma; Epimedii Folium; Morindae Officinalis Radix; Angelicae Sinensis Radix; Anemarrhenae Rhizoma; Phellodendri Cortex; Bupleuri Radix; Paeoniae Radix Alba; Atractylodis Macrocephalae Rhizoma; Poria; Menthae Haplocalycis Herba; Glycyrrhizae Radix Preparata;Zingiberis Rhizoma*	MS+RS mice model	p.o. 16.71 g·kg^-1^ for 90 days	NLRP3 inflammasome-associated microglial inflammatory regulation and synaptic remodeling	PSD-95; Syn↑ IL-1β;IL-18; NLRP3;Caspase-1;ASC↓	Associative evidence	(96)
Anmeidan	*Semen Ziziphi Spinosae; Radix et Rhizoma Ginseng; Ophiopogonis Radix; Poria; Angelicae Sinensis Radix; Salviae Miltiorrhizae Radix et Rhizoma; Acori Tatarinowii Rhizoma; Schisandrae Chinensis Fructus; Glycyrrhizae Radix et Rhizoma*	CUMS rat model	i.g. 1.5, 3 g·kg^-1^ for 4 weeks	BDNF/CaMKII-associated synaptic resilience and inflammatory stress regulation	IL-6;TNF-α;IL-1*β*; Arc↓ PSD-95; BDNF;p-CaMKII/CaMKII; Ca^2+^↑	Indirect evidence	(97)
Zhizichi Decoction	*Gardeniae Fructus; Sojae Semen Praeparatum*	CUMS rat model	i.g. 3.25, 6.50 g·kg^-1^ for 3 weeks	Iba-1/NLRP3 inflammasome-associated microglial inflammatory regulation and synaptic remodeling	Synapsin-1;PSD-95↑ NLRP3;Caspase-1;IL-1β; IL-18; Cleaved GSDMD; Iba-1↓	Associative evidence	(98)
Chaijin Jieyu Anshen Tablets	*Bupleurum chinense DC; Hypericum perforatum L.; Paeonia lactiflora Pall.; Curcumae Radix; Citrus medica L.* var. *sarcodactylis Swingle; Poria cum radix pini; Ziziphi Spinosae Semen; Valeriana officinalis L.*	CORT+LPS cell model	10% body weight fraction	Glutamatergic signaling and synaptic resilience pathways associated with neuronal vulnerability	CaMKII;Cofilin↑ MK2;GRIN2A; GRIN2B;c-FOS;Glu↓	Indirect evidence	(99)
Chaijin Jieyu Anshen Tablets	*Bupleurum chinense DC; Hypericum perforatum L.; Paeonia lactiflora Pall.; Curcumae Radix; Citrus medica L.* var. *sarcodactylis Swingle; Poria cum radix pini; Ziziphi Spinosae Semen; Valeriana officinalis L.*	social isolation combined with CUMS rat model	i.g. 2.84, 5.67, 11.34 g·kg^-1^ for 4 weeks	Glutamatergic signaling and cytoskeletal remodeling pathways associated with synaptic resilience	Cofilin; CaMKII↑ MK2;GRIN2A; GRIN2B↓	Indirect evidence	(100)
Chaijin Jieyu Anshen Formula	*Bupleuri Radix; Hyperici Perforati Herba; Paeoniae Radix Alba; Anemarrhenae Rhizoma*	PCPA combined with CUMS rat model	i.g. 2.7, 5.4, 10.8 g·kg^-1^ for 4 weeks	Epigenetic regulation of synaptic resilience pathways (HDAC5/MEF2 signaling)	HDAC5↓ MEF2; PSD-95;SYN1;ARC↑	Indirect evidence	(101)
Sinisan	Chaihu (Radix Bupleuri), *Shaoyao* (Radix Paeoniae Alba), *Zhishi* (Fructus Aurantii Immaturus) and Gancao (Radix et Rhizoma Glycyrrhizae).	MS rat model	i.g. 2.5, 5.0, 10.0 g·kg^-1^ for 30 days	Mitochondrial homeostasis-related synaptic resilience pathways	PSD-95;SYN;ATP;Mfn2↑ Drp1;Fis1↓	Indirect evidence	(102)
Chaihu-Baishao	Chaihu and Baishao	chronic social defeat stress (CSDS) mouse model	i.g. 7.5, 15, 30 g·kg^-1^ for 10 days	Glutamatergic synaptic resilience pathways associated with synaptic remodeling	PSD-95;SYN↑ α2δ-1;NR2B↓	Indirect evidence	(103)
Zuogui Jiangtang Jieyu Formula	*Rehmanniae Radix Praeparata; Corni Fructus; Lycii Fructus; Cuscutae Semen; Hyperici Perforati Herba; Salviae Miltiorrhizae Radix et Rhizoma; Moutan Cortex; Curcumae Longae Rhizoma; Achyranthis Bidentatae Radix; Eucommiae Cortex; Astragali Radix*	social isolation combined with CUMS rat model	i.g. 10.26 g·kg^-1^ for 14 days	TREM2/C1q complement-associated microglial synaptic remodeling pathway	TREM2; PSD-95↑ C1q↓	Associative evidence	(104)
Zhuangu Zhitong Jieyu Formula	*Psoraleae Fructus; Epimedii Folium; Lycii Fructus; Ligustri Lucidi Fructus; Cibotii Rhizoma; Drynariae Rhizoma; Curcumae Longae Rhizoma; Cyathulae Radix; Hyperici Perforati Herba; Radix et Rhizoma Ginseng; Bupleuri Radix*	synovial cell inflammation model	10% body weight fraction	CX3CL1/CX3CR1-associated neuroimmune signaling and synaptic remodeling pathways	Occludin;ZO-1↑ TNF-α;RF;FKN; CX3CR1; A2AR; NR2A;NR2B↓	Associative evidence	(105)
Baishile Capsule	*Hypericum perforatum L.;Panax ginseng C. A. Mey;Curcuma longa L.*	social isolation combined with CUMS rat model	i.g. 0.72, 2.88g·kg^-1^ for 4 weeks	Neurotrophic and inflammatory stress regulation pathways associated with synaptic resilience	Igf2;Rpl36↑ TNF-α;IL-6↓	Indirect evidence	(106)
Baishile Capsule	*Panax ginseng C. A. Mey; Hypericum perforatum L.*; Curcuma longa L.	social isolation combined with CUMS rat model	i.g. 2.88 g·kg^-1^ for 3 weeks	Neurogenesis-associated pathways influencing synaptic resilience and circuit remodeling	BrdU/GABA-B;BrdU/c-fos; BrdU/GAP-43;BrdU/MAP-2; CXCR4↑	Indirect evidence	(107)
Jiannao Jieyu Formula	*Rehmanniae Radix Praeparata; Paeoniae Radix Alba; Croci Stigma; Corni Fructus; Dioscoreae Rhizoma; Acori Tatarinowii Rhizoma; Curcumae Longae Rhizoma; Hyperici Perforati Herba; Glycyrrhizae Radix et Rhizoma Praeparata cum Melle*	CSVDD rat model	i.g. 7.02, 14.04, 28.08 g·kg^-1^ for 3 weeks	BDNF-associated synaptic resilience and inflammatory stress regulation pathways	NO; BDNF; Syn; PSD-95↑ ET-1; Glu; IL-6;TNF-α↓	Indirect evidence	(108)
*Gongjin-Dan*	*Rehmannia glutinosa, Cervi pantiorichum, Angelicae Radix, Dioscorea polystachya, Cornus officinalis, Wolfiporia extensa, Alisma canaliculatum, Moutan Cortex* Radicis, *Musk*, and *Aquilaria agallocha* Roxburgh.	Primary Rat Cortical Neurons	10, 25, 50 μg·mL^-1^	Oxidative stress regulation and neurotrophic pathways associated with synaptic resilience	Nrf2;Sirt1;BDNF;NGF; Synapsin-1↑ H_2_O_2_↓	Indirect evidence	(109)

#### 5.1.1 Enhancement of synaptic resilience through the BDNF/TrkB neurotrophic signaling pathway

The BDNF/TrkB pathway is a critical regulator of synaptic maintenance, structural stability, and stress resilience. In depression, impaired BDNF/TrkB signaling compromises synaptic maintenance and structural stability, which may increase neuronal vulnerability to stress-induced synaptic remodeling and excessive elimination. TCM formulas may enhance BDNF-related neurotrophic signaling through multiple targets, thereby promoting synaptic resilience and structural stability.

In the Chronic Unpredictable Mild Stress (CUMS) rat model, Xiaoyao San activates the BDNF/TrkB signaling pathway and upregulates PSD-95 expression, thereby enhancing synaptic strength and growth in the hippocampal CA3 region (84). By strengthening synaptic integrity, Xiaoyao San may reduce susceptibility to stress-induced pathological synaptic impairment and pruning-associated vulnerability. In addition, Xiaoyao San has been reported to regulate gut microbiota dysbiosis and inhibit microbiota-associated peripheral and prefrontal complement C3 activation, suggesting a potential influence on complement-related mechanisms involved in microglia-associated synaptic remodeling (85). Mume Pill activates the BDNF/TrkB/PLCγ1 pathway and its downstream CaMK2/CREB signaling in the hippocampal dentate gyrus (DG). These effects may enhance synaptic stability under stress conditions and reduce vulnerability to pruning-associated synaptic dysfunction (86). Wenxiao San and Wenyang Jieyu Fang promote neurogenesis and synaptic structural remodeling by regulating the p-TrkB/ERK/CREB and Akt/mTOR signaling axes, respectively. These effects may improve neural circuit dysfunction associated with stress-induced synaptic impairment and enhance neuronal resilience (87, 88). Baihe Dihuang Tang inhibits MAOA expression and reduces the oxidative degradation of 5-HT, thereby increasing 5-HT bioavailability. This subsequently enhances BDNF/TrkB signaling and the expression of synaptic plasticity-related proteins, enhancing synaptic resilience under stress-related structural vulnerability (89). Xiaoyao Kangai Jieyu Recipe regulates PI3K/Akt signaling to enhance neurotrophic support and promote the repair of hippocampal synaptic plasticity, thereby improving stress-induced synaptic dysfunction and potentially reducing susceptibility to synaptic structural impairment (90).

Collectively, these findings provide indirect evidence that TCM formulas may influence pruning-associated vulnerability by enhancing neurotrophic support and synaptic resilience. However, whether BDNF/TrkB activation directly alters microglial synaptic engulfment remains to be determined.

#### 5.1.2 Modulation of neuroinflammatory responses and microglial activation states

In depression, excessive microglial activation is associated with inflammatory responses and dysregulated synaptic remodeling, which may contribute to increased synaptic vulnerability and abnormal synaptic elimination. TCM formulas may modulate key inflammatory signaling pathways, including NF-κB and NLRP3 signaling pathways, which are implicated in microglial activation and synaptic dysfunction. Through these mechanisms, TCM formulas may alleviate neuroinflammatory disturbances and preserve synaptic integrity, thereby potentially reducing vulnerability to inflammation-associated synaptic remodeling.

Regarding inflammasome-related inflammatory signaling, Zhizi Houpotang and Compound Chaijin Jieyu Fang have been reported to inhibit NLRP3 inflammasome activation and GSDMD-mediated pyroptosis through regulation of the P2RX7/Panx1 and TLR4/NLRP3 signaling axes, respectively. These effects reduce the release of pro-inflammatory cytokines, including IL-1β and IL-18, and improve inflammation-associated synaptic alterations, as reflected by restored PSD-95 and Synapsin-1 expression and preserved dendritic spine density and morphology (91, 92). In terms of reshaping the immune microenvironment balance, Xingnao Jieyu Decoction and Suanzaoren Tang function by multi-directionally regulating the immune factor network. Xingnao Jieyu Decoction inhibits the NF-κB/IκB-α pathway; while downregulating IL-6 and TNF-α, it restores levels of anti-inflammatory factors like IL-4 and IL-10. This immunomodulatory effect may contribute to the transition of microglia toward a more homeostatic phenotype (93, 94). Jiawei Buyang Huanwu Tang, Wenyang Jieyu Fang, and Anmei Dan have been reported to modulate microglial activation associated with inflammatory responses. Jiawei Buyang Huanwu Tang has been reported to attenuate microglial overactivation and reduce the expression of inflammatory and phagocytosis-associated markers (95). Wenyang Jieyu Prescription alleviates neuroinflammation by regulating the NLRP3 pathway and regulating inflammatory signaling and neurotransmitter metabolism (96). Anmei Dan protects synaptic structures under inflammatory conditions by inhibiting pro-inflammatory cytokines and enhancing neurotrophic support to mitigate neuronal damage (97). Additionally, Zhizi Chi Tang improves synaptic injury by inhibiting the activation of the hippocampal NLRP3 inflammasome and reducing the release of IL-1β and IL-18, which may reduce inflammatory signaling associated with microglial activation and phagocytosis-related responses (98).

Taken together, these findings provide associative evidence that TCM formulas may influence microglial activation states and inflammatory environments related to synaptic remodeling. However, whether these anti-inflammatory effects directly alter microglial synaptic engulfment remains insufficiently investigated.

#### 5.1.3 Regulation of glutamatergic signaling and mitochondrial homeostasis associated with synaptic vulnerability

Excessive glutamatergic activity and mitochondrial energy deficits can compromise synaptic stability, potentially increasing neuronal susceptibility to stress-induced synaptic remodeling and elimination. TCM formulas may regulate glutamatergic signaling and mitochondrial dynamics, thereby enhancing synaptic resilience under pathological conditions. These effects may help maintain synaptic homeostasis within pathological microenvironments and reduce vulnerability to pruning-associated synaptic dysfunction.

Chaijin Jieyu Anshen tablets have been reported to improve glutamatergic signaling abnormalities and synaptic deficits by modulating neuronal signal transduction networks. They reduce excessive glutamatergic activation in the anterior cingulate cortex (ACC) and hippocampus, downregulating GRIN2A, GRIN2B, and MK2 proteins while upregulating CaMKII and Cofilin. By modulating the NR/CaMKII and MK2/Cofilin signaling axes, they stabilize the cytoskeleton and reduce excitotoxicity-associated synaptic impairment. Furthermore, this formula regulates abnormalities in the ACC-vHPC neural circuit and promotes the expression of PSD-95 and SYN-1 by reducing HDAC5 expression, which relieves the inhibition on the transcription factor MEF2C. This improvement in synaptic structural integrity may enhance synaptic resilience and reduce susceptibility to stress-associated synaptic remodeling (99–101). In contrast, Sini San may reduce pruning-associated synaptic vulnerability through regulation of neuronal energy metabolism. Excessive mitochondrial fission is associated with synaptic dysfunction and structural impairment. Sini San corrects mitochondrial dynamics imbalance by upregulating the mitochondrial fusion protein Mfn2 and inhibiting the fission proteins Drp1 and Fis1, thereby elevating ATP levels in the hippocampus. This improved bioenergetic state supports the maintenance of synaptic structure and function under stress conditions. Subsequently, it promotes PSD-95 and SYN expression. Simultaneously, the formula restores CaSR expression in the dorsal raphe nucleus (DRN), effectively alleviating synaptic loss in this key nucleus. By improving neuronal metabolic homeostasis, Sini San may reduce neuronal stress and decrease susceptibility to stress-associated synaptic impairment (102).

From the perspective of current evidence, these findings mainly provide indirect support that TCM formulas may enhance synaptic resilience by improving glutamatergic balance and mitochondrial function. Whether these metabolic and neuronal effects directly influence microglial synaptic engulfment requires further investigation.

#### 5.1.4 Preservation of synaptic structure and neuronal integrity associated with pruning vulnerability

In depression, disruption of synaptic scaffolding and deterioration of the local neural microenvironment may increase synaptic vulnerability and influence microglial responses associated with synaptic remodeling. TCM formulas may enhance synaptic resilience by stabilizing synaptic scaffold proteins, regulating neuronal survival signaling, and maintaining the neurovascular unit. These effects may help preserve synaptic integrity under pathological conditions and reduce susceptibility to pruning-associated synaptic vulnerability.

Regarding the regulation of synaptic scaffold and receptor interactions, the Chaihu-Baishao herb pair alleviates structural damage to hippocampal neurons by downregulating the aberrant interaction between α2δ-1 and NR2B, thereby stabilizing the postsynaptic membrane structure and preventing synaptic protein degradation. This preservation of synaptic ultrastructure may reduce structural vulnerability associated with excessive synaptic remodeling (103). Zuogui Jiangtang Jieyu Formula represents one of the relatively relevant examples linking TCM intervention with pruning-associated signaling pathways. It upregulates TREM2 expression and regulates complement-associated synaptic abnormalities through the C1q/PSD-95 signaling pathway in diabetic depression rats, suggesting potential involvement in microglia-synapse interactions (104). However, direct assessment of microglial synaptic engulfment was not performed; therefore, this evidence should be considered associative rather than direct evidence of altered pruning. By regulating the FKN/CX3CR1 signaling pathway, Zhuanggu Zhitong Jieyu Fang may reduce microglial chemotactic activation and inflammatory responses associated with synaptic dysfunction, thereby improving the synaptic microenvironment (105). Regarding the promotion of neurogenesis and structural reconstruction, Baishile Capsules and Naojieyu Fang focus on reducing synaptic vulnerability by enhancing neuronal survival capacity. These effects mainly reflect indirect evidence of enhanced neuronal resilience, as their influence on microglial pruning activity has not been directly examined. Baishile Capsules regulate neuronal survival via the Igf2/MAPK and insulin signaling pathways and reduce apoptosis through Rpl36, while simultaneously promoting the expression of regenerative proteins such as GAP-43 and MAP-2 to promote neuronal maturation and structural remodeling (106, 107). Jiannao Jieyu Formula promotes the recovery of hippocampal synaptic plasticity by elevating neurocellular nutritional levels (108). In the realm of antioxidant and gene regulation, Gongjin-Dan activates the Sirtuin1 signaling pathway to mitigate oxidative stress damage to neurons and inhibit neuronal death. Simultaneously, it upregulates NGF, BDNF, and regeneration-related genes to promote new axon extension and synaptogenesis, thereby reducing oxidative stress-associated synaptic impairment and preserving synaptic integrity (109).

Based on the available evidence, TCM formulas involving complement-related pathways, such as TREM2/C1q regulation, provide associative evidence linking TCM interventions with pruning-associated mechanisms. In contrast, studies focusing on synaptic proteins, neuronal survival, and oxidative stress mainly provide indirect evidence by demonstrating enhanced synaptic resilience rather than direct modulation of microglial synaptic engulfment.

### 5.2 Single Chinese herbs

Single Chinese herbs and their extracts may influence the microglia-synapse interface through regulation of glial activation, inflammatory responses, and synaptic resilience-related processes. They may regulate glial activation states and pathways related to inflammatory and phagocytosis-associated responses, while also improving the cerebral immune microenvironment through mechanisms such as the gut-brain axis and the renin-angiotensin system (RAS). In addition, many studies report that these interventions increase the expression of synaptic-associated proteins, thereby enhancing synaptic resilience and potentially reducing susceptibility to stress-related synaptic impairment. The relevant evidence is summarized in **Table 2**.

**Table 2:** Summary of single Chinese herbs potentially influencing pruning-associated pathways and synaptic plasticity in depression models.

Name	Origin	Extract	Animal/cell model	Dose/duration of administration	Pruning-associated pathway	Effects and mechanisms	Evidence level	References
Inulae Flos	The flower of *Inula japonica* Thunb	ethanol	CRS mouse model	p.o. 0, 100, 300mg·kg^-1^ for 2 weeks	Stress-response and neuroimmune pathways associated with synaptic resilience	SYP;GR↑ iNOS;IL-6;CORT↓	Indirect evidence	(110)
*Saccharina japonica*	Not specified	ethanol	DSS mice model	p.o. 1, 2, 4g·kg^-1^ for 2 weeks	NF-κB/NLRP3 inflammasome-associated inflammatory regulation related to microglial activation	SOD↑ MDA;NF-κB;p65;NLRP3;ASC;Caspase-1↓	Associative evidence	(111)
Saffron	*Crocus sativus L.*	ethanol	CUMS rat model	i.g. 80, 160mg·kg^-1^ for 1 week	BDNF-associated synaptic resilience and neuroinflammatory regulation pathways	SYP;PSD-95;BDNF↑ Agt;Ang-II;IL-6;TNF-α↓	Indirect evidence	(112)
*Astragalus membranaceus*	Not specified	Not specified	Chronic sleep deprivation model	i.g. 2.5, 25 g·kg^-1^ for 1 week	Neuroimmune regulation and activity-dependent synaptic resilience pathways	Nr4a1;Fos;Egr4↑ TNF-α; IL-6; IL-1β; MCP-1↓	Indirect evidence	(113)
pine needle	Pinus densiflora Siebold & Zucc.	hot water	LPS-induced neuroinflammation mouse model	p.o. 50 mg·kg^-1^ for 1 week	Neuroinflammatory regulation and BDNF-associated synaptic resilience pathways	BDNF↑ TNF-α;IL-1β;apelin↓	Indirect evidence	(114)
Persimmon leaf	*Diospyros kaki Thunb.*	Not specified	CSDS mouse model	p.o. 30, 60 mg·kg^-1^ for 10 days	cAMP/PKA/CREB-BDNF-associated synaptic resilience pathways	PSD-95; Synapsin-1; cAMP/PKA/CREB; BDNF↑	Indirect evidence	(115)
*Fructus Aurantii*	*Citrus aurantium L.*	water	LPS-induced depression model	Single i.p. 9.18 mg·kg^-1^	Neuroinflammatory regulation and synaptic resilience pathways	PSD-95; Synapsin-1↑ TNF-α;IL-1β;Caspase-4↓	Indirect evidence	(116)
Qingyangshen	*C. otophyllum* C.K. Schneid	ethanol	CSDS mouse model	p.o. 25, 50, 100 mg·kg^-1^	Neurogenesis-associated synaptic resilience and structural remodeling pathways	SYN;PSD-95; DCX↑	Indirect evidence	(117)

#### 5.2.1 Regulation of glial activation and inflammatory pathways associated with pruning susceptibility

Inula Flos (IF) has been reported to attenuate stress-induced activation of microglia and astrocytes. By lowering plasma corticosterone (CORT) levels and restoring hypothalamic glucocorticoid receptor expression, it attenuates stress-induced glial activation, thereby improving synaptic plasticity (110). The ethanol extract of Saccharina japonica (SJE) promotes a more homeostatic microglial phenotype and reduces markers associated with pro-phagocytic activation through regulation of the TLR4-NLRP3 signaling pathway. In addition, this extract reduces oxidative stress and apoptosis, thereby contributing to maintenance of the synaptic microenvironment (111). Xihonghua (Saffron Stigma) modulates the renin-angiotensin system (RAS) by regulating Agt expression in the nucleus accumbens. This regulation may reduce neuroinflammation and oxidative stress, while increasing the expression of SYP, PSD-95, and BDNF, thereby supporting synaptic remodeling and resilience under inflammatory conditions (112).

These findings provide associative evidence that single Chinese herbs may influence microglial activation states and inflammatory environments related to pruning susceptibility. However, direct evidence demonstrating altered microglial synaptic engulfment following these interventions remains limited.

#### 5.2.2 Regulation of the gut-brain axis and systemic immune environment

Astragalus (AST) has been reported to modulate sleep deprivation-induced gut microbiota dysbiosis by reducing potentially harmful bacterial alterations and enriching beneficial bacteria, thereby promoting the production of neuroprotective metabolites. Concurrently, AST may improve intestinal barrier integrity through regulation of ZO-1 expression and reduce systemic inflammatory cytokines, including TNF-α, IL-6, and IL-1β. These effects may reduce peripheral inflammatory signals reaching the central nervous system, contribute to the normalization of hippocampal and amygdala neural activity, and increase the expression of neuroplasticity-related genes such as Nr4a1 and Fos, thereby supporting synaptic function (113).

These findings mainly represent indirect evidence, suggesting that modulation of the gut-brain axis and systemic immune environment may influence synaptic resilience and microglial-related processes. However, direct evidence linking gut microbiota regulation by AST to altered microglial synaptic engulfment remains unavailable.

#### 5.2.3 Enhancement of synaptic structural resilience and neuronal plasticity

Hot water extracts of pine needles (PN) have been reported to activate the Apelin signaling pathway, increase hippocampal BDNF levels, attenuate neuroinflammatory responses, and promote neurogenesis. This enhancement of neurotrophic support may improve synaptic resilience and reduce susceptibility to stress-associated synaptic impairment (114). Persimmon Leaf Extract (PLE) restores CSDS-induced reductions in dendritic complexity and spine density by regulating 5-HT signaling and modulating the cAMP/CREB/BDNF pathway, thereby enhancing synaptic structural stability and neuronal resilience (115). Zhiqiao (MH) protects hippocampal synaptic plasticity by maintaining glial support for neuronal homeostasis and inhibiting Caspase-4 activation, thereby reducing synaptic impairment associated with apoptotic signaling (116). Qingyangshen (QYS) enhances hippocampal neuroplasticity by upregulating synapse-associated proteins (SAPs), thereby supporting synaptic structural resilience (117).

### 5.3 Bioactive compounds derived from traditional Chinese medicinal sources

The biological effects of TCM formulas are partly mediated by bioactive compounds derived from their constituent medicinal materials. These bioactive compounds represent important molecular mediators that may contribute to the effects of traditional medicinal interventions on the microglia-synapse interface in depression. By regulating neurotrophic, inflammatory, and structural pathways, these compounds may influence biological processes associated with pruning susceptibility and contribute to restoration of synaptic homeostasis. This section summarizes current evidence from representative bioactive compounds and discusses their potential mechanisms in regulating synaptic resilience and microglia-associated processes in depression. In this review, bioactive compounds are considered TCM-derived compounds when they are isolated from, enriched in, or investigated in the context of traditionally used Chinese medicinal materials or TCM formulations. Although some compounds, such as EGCG, quercetin, lycopene, and rosmarinic acid, are also distributed in dietary plants or other natural sources, the studies included in this section were selected based on their association with TCM sources and their pharmacological investigations within the context of Chinese medicinal applications. The representative bioactive compounds are summarized in **Table 3**.

**Table 3:** Summary of bioactive compounds derived from Chinese medicinal sources associated with pruning-related pathways and synaptic plasticity in depression models.

Name	Structure	Animal/cell model	Dose/duration of administration	Pruning-associated pathway	Effects and mechanisms	Evidence level	References
Ginsenoside Rb1	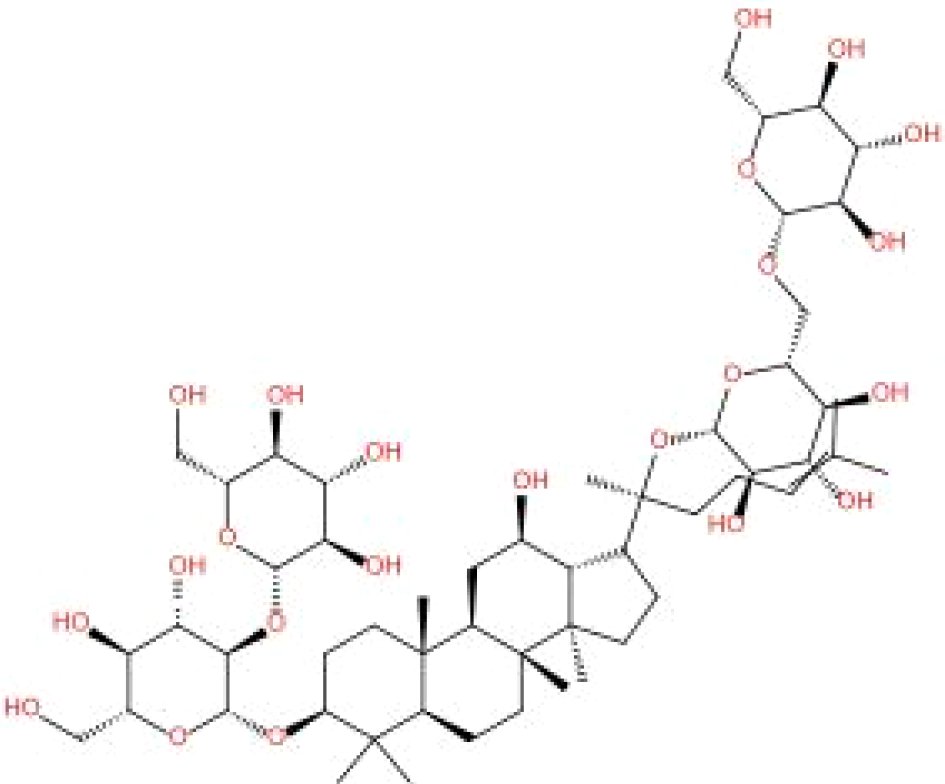	CUMS mouse model	i.g. 0.9 mM for 35 days	BDNF/TrkB/Akt-associated synaptic resilience and glutamatergic remodeling pathways	PSD-95; GAP-43; MAP-2; NR2A; NR2B; GluR1; Ca^2+^/CaMKII; BDNF; Akt; TrkB↑	Indirect evidence	(118)
Rosmarinic acid	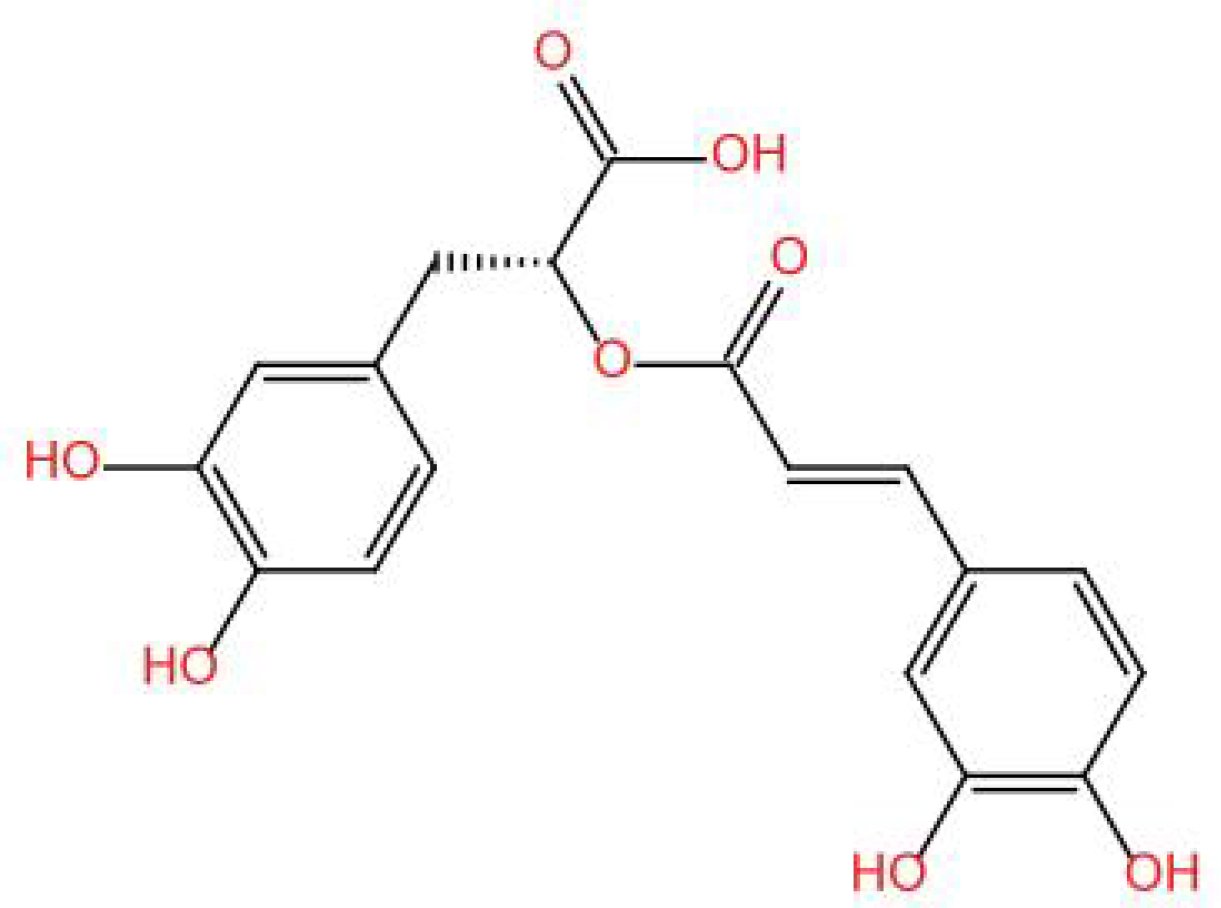	chronic corticosterone (CORT)-induced depression model (for 21 days)	p.o. 10 mg·kg^-1^ for 3 weeks	BDNF/CREB-associated synaptic resilience pathways	Synaptophysin; PSD-95;p-CREB/CREB; BDNF ↑	Indirect evidence	(119)
Engeletin	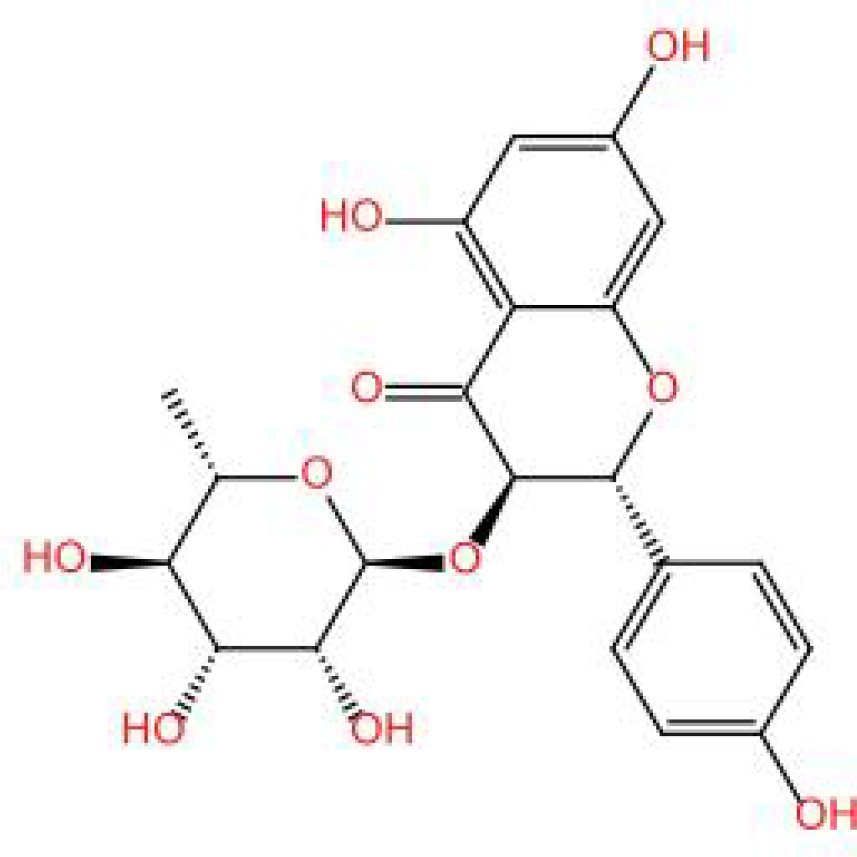	CRS mouse model	i.g. 2.5,5,10,20 mg·kg^-1^ for 4 weeks	BDNF-associated synaptic resilience and glutamatergic remodeling pathways	GluR1;PSD-95;BDNF;Synapsin-1 ↑	Indirect evidence	(120)
Lycopene	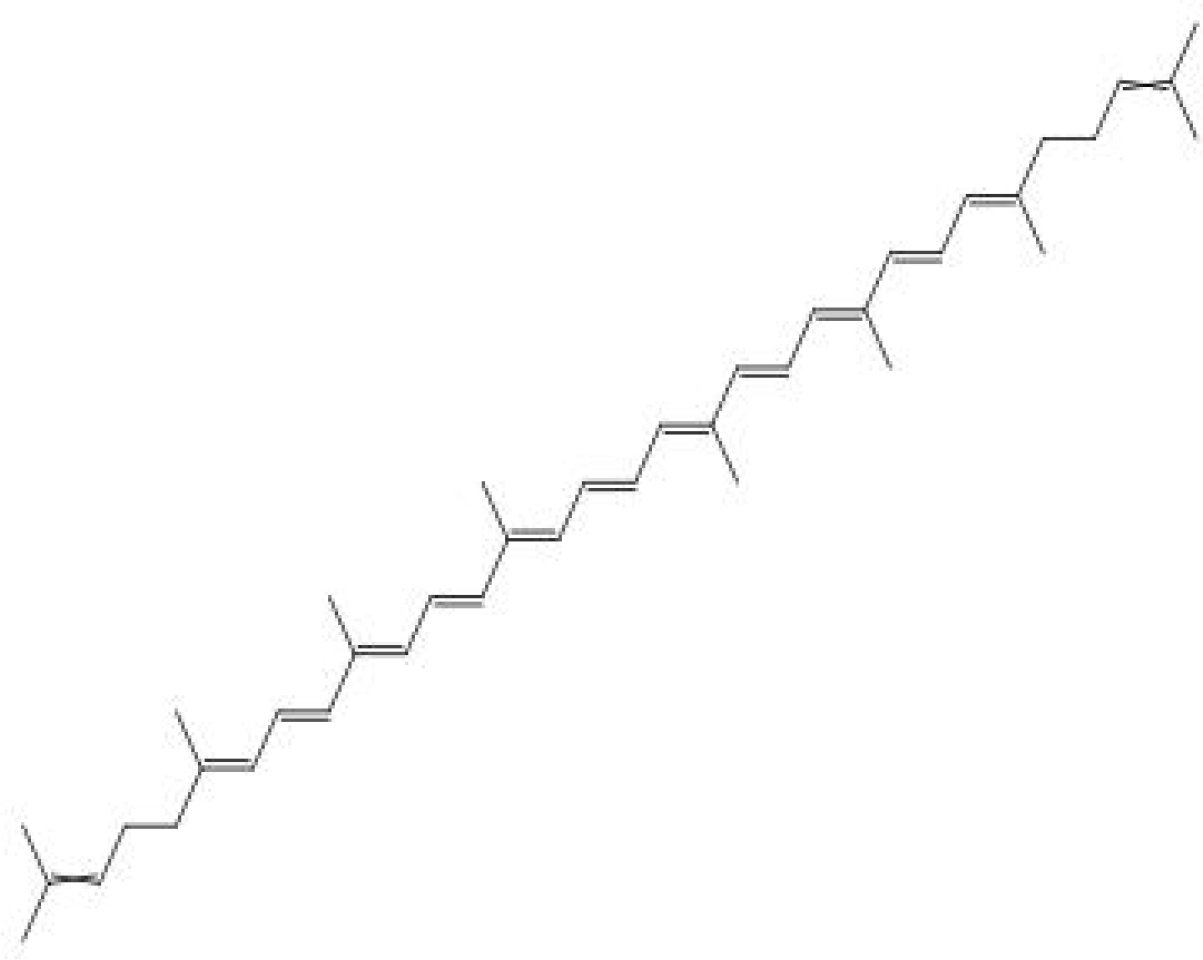	CSDS mouse model	p.o. 20 mg·kg^-1^ for 10 days	BDNF/TrkB-associated synaptic resilience pathways	Syn;PSD-95; BDNF;TrkB;p-TrkB ↑	Indirect evidence	(121)
Kaempferol-3-O-sophoroside (PCS-1)	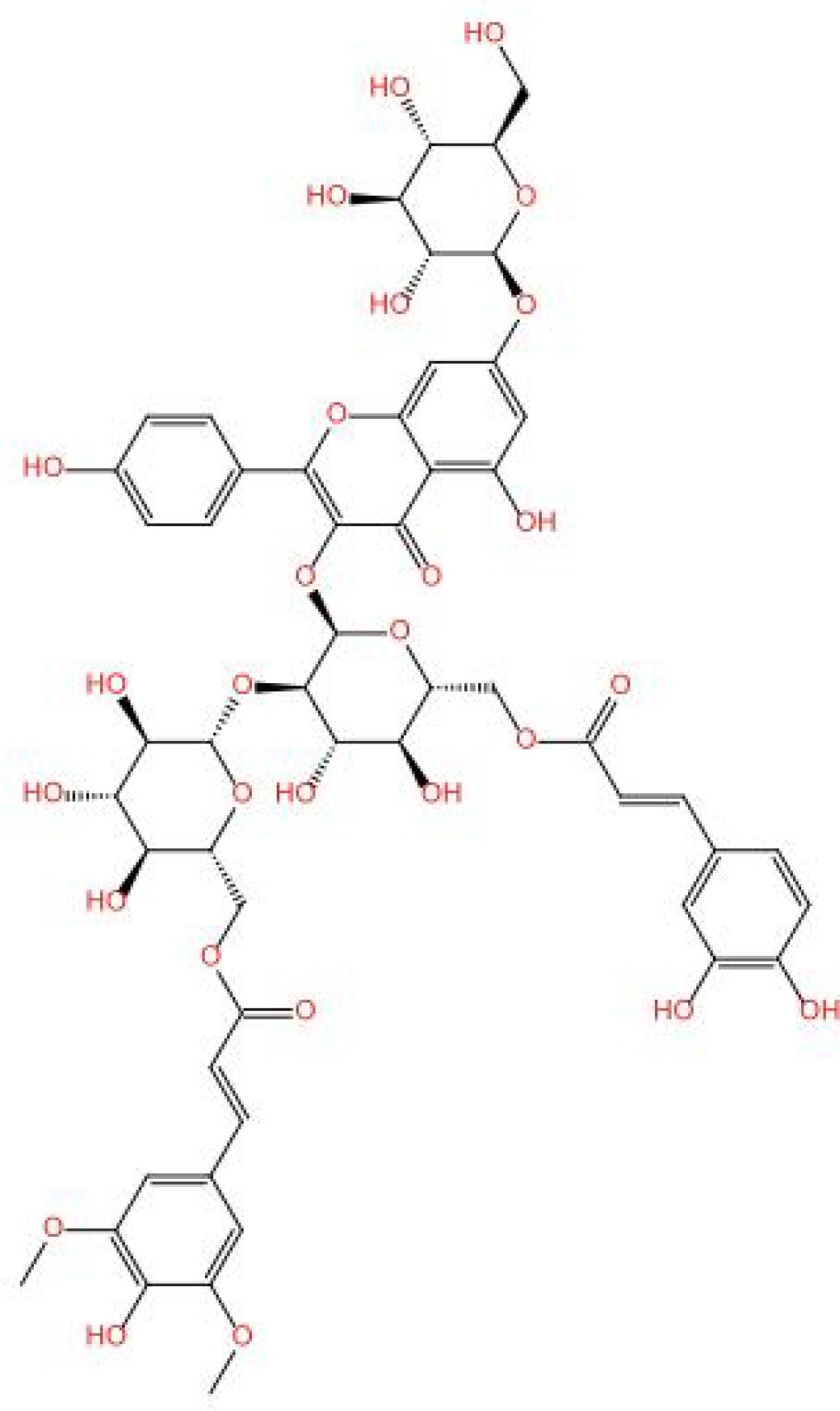	CORT mouse model	i.g. 10,20 mg·kg^−1^ for 20 days	Mitochondrial homeostasis and BDNF-associated neuronal resilience pathways	Bcl-2/Bax;caspase-9;caspase-3; ROS;MDA↓ SOD;BDNF;MAP-2;Tau↑	Indirect evidence	(122)
Epigallocatechin-3-gallate	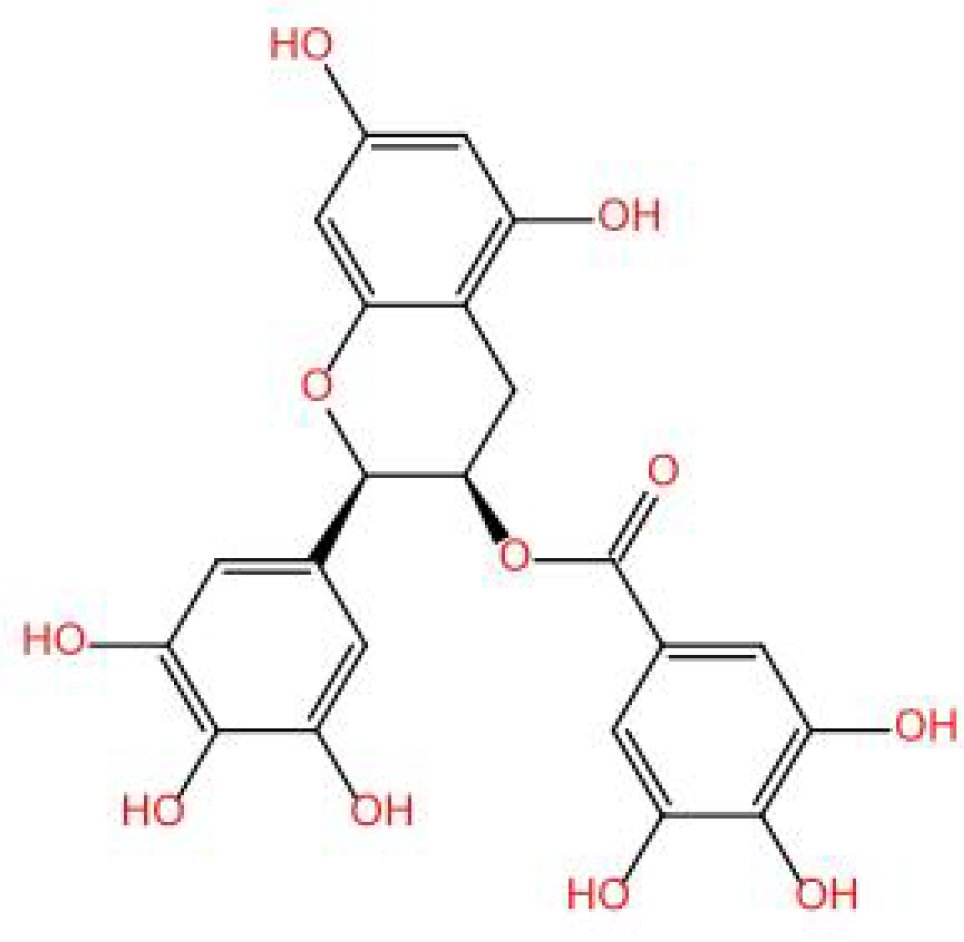	CUMS mouse model	b.w. 200 mg·kg^−1^ for 2 weeks	BDNF-associated neurotrophic and inflammatory stress regulation pathways	IL-1β↓ BDNF↑	Indirect evidence	(123)
Baicalin	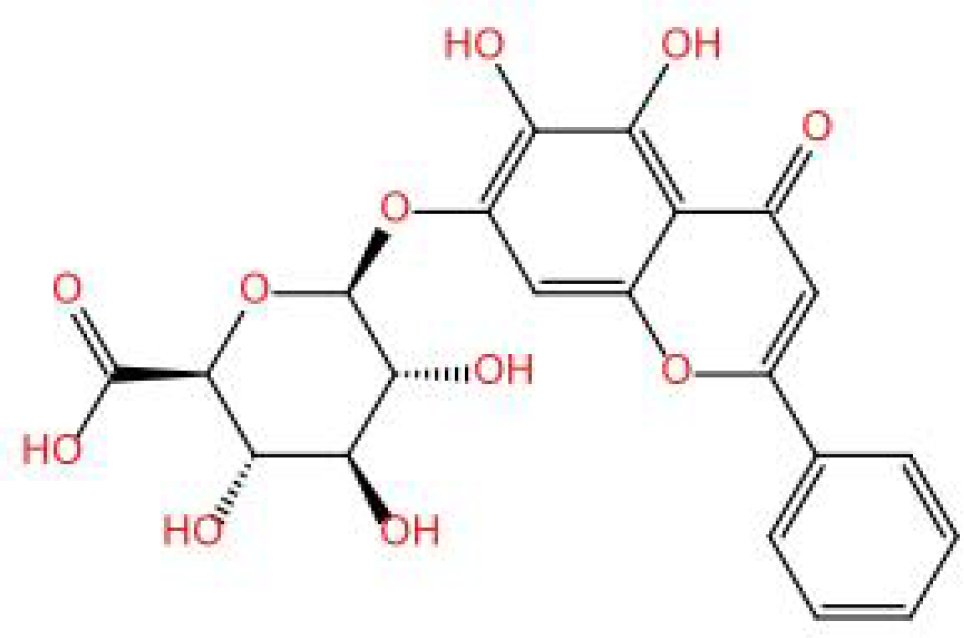	CRS mouse model	i.g. 10 or 40 mg·kg^−1^ for 4 weeks	Oxidative stress regulation and BDNF-associated neuronal resilience pathways	ROS;ROS/H_2_O_2_ ↓ BDNF↑	Indirect evidence	(124)
Quercetin	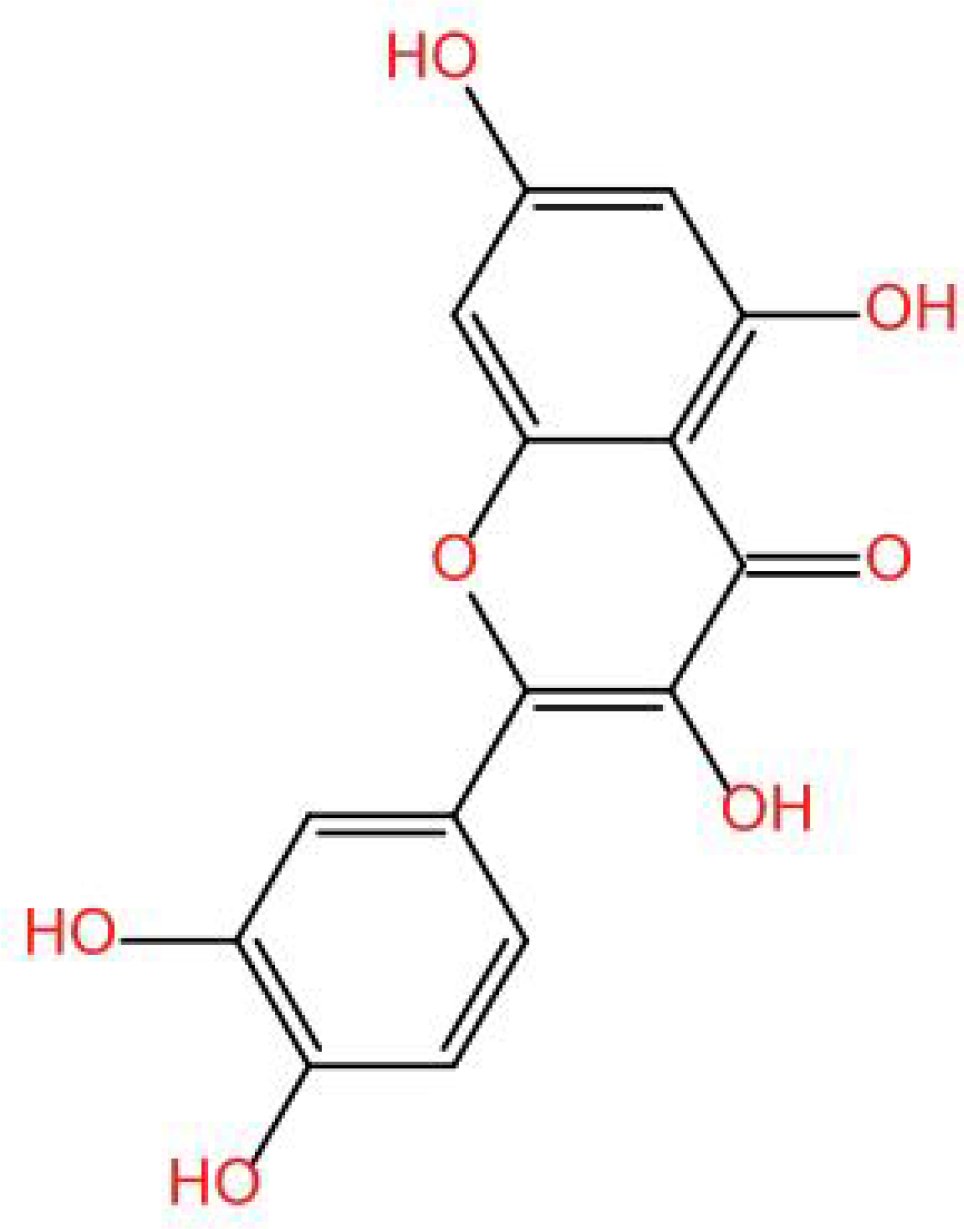	CUMS mouse model	i.g. 30 mg·kg^−1^ for 4 weeks	BDNF-associated synaptic resilience and neuroinflammatory regulation pathways	TNF-α;IL-1β↓ PSD-95;Arc;BDNF↑	Indirect evidence	(125)
Ginsenoside Rg3	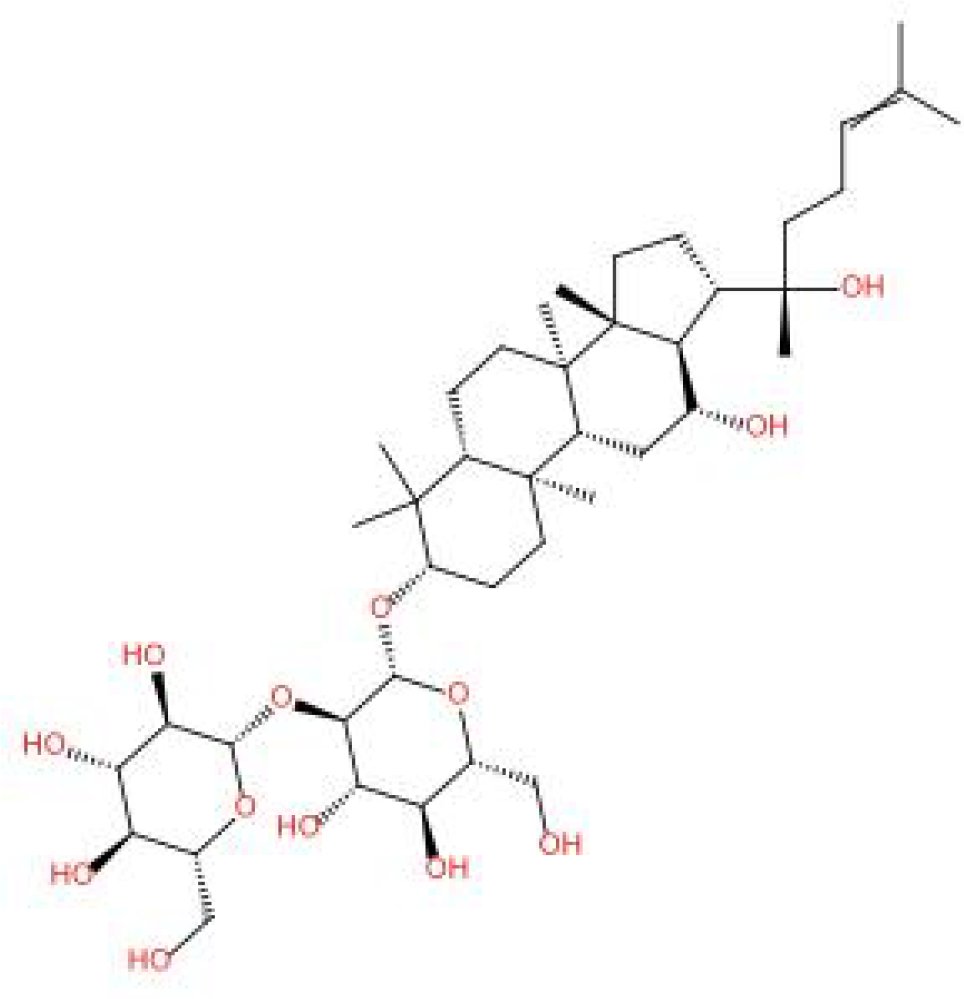	CRS mouse model	i.g. 10,20,40 mg·kg^−1^ for 8 weeks	C1q-associated complement pathway and synaptic remodeling	PSD-95;SYN;Bcl-2↑ Bax;C1q↓	Associative evidence	(126)
Emodin	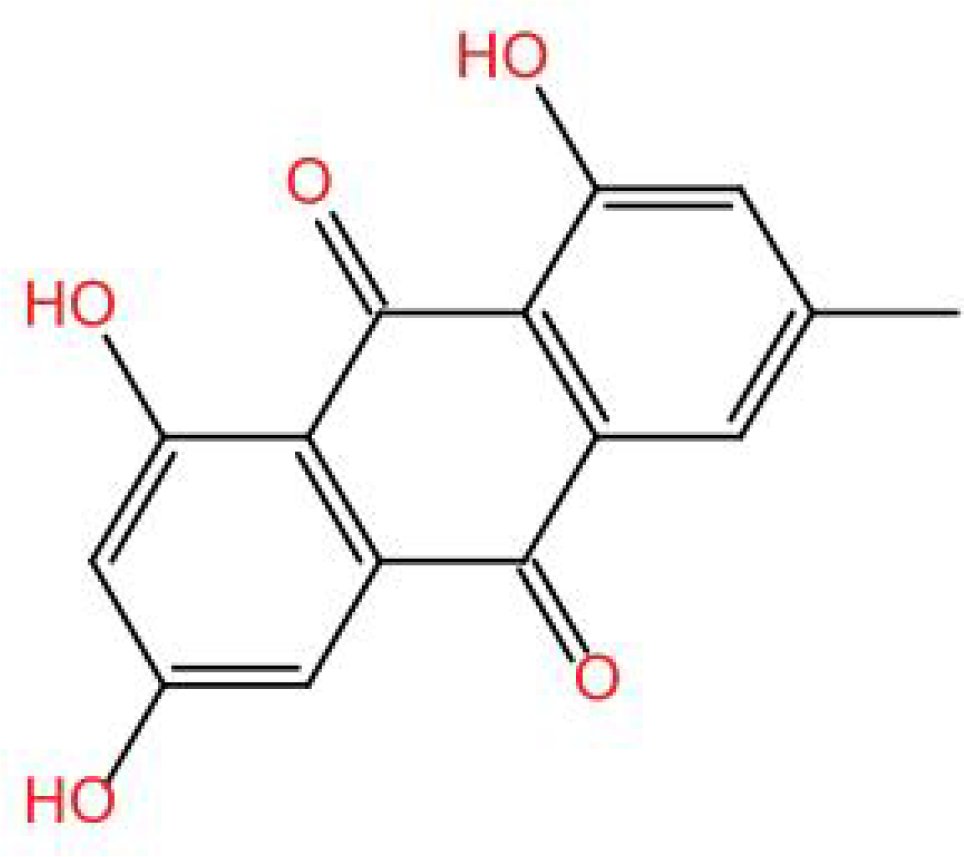	CUMS mouse model	i.g. 80 mg·kg^−1^ for 2 weeks	NF-κB/Nrf2-associated inflammatory and oxidative stress regulation pathways related to synaptic resilience	TNF-α;IL-1β;NF-κB↓ Fga;Fgb;Fgg;Ahsg;Col1a1; Nrf2;Anxa2↑	Indirect evidence	(127)
Berberine	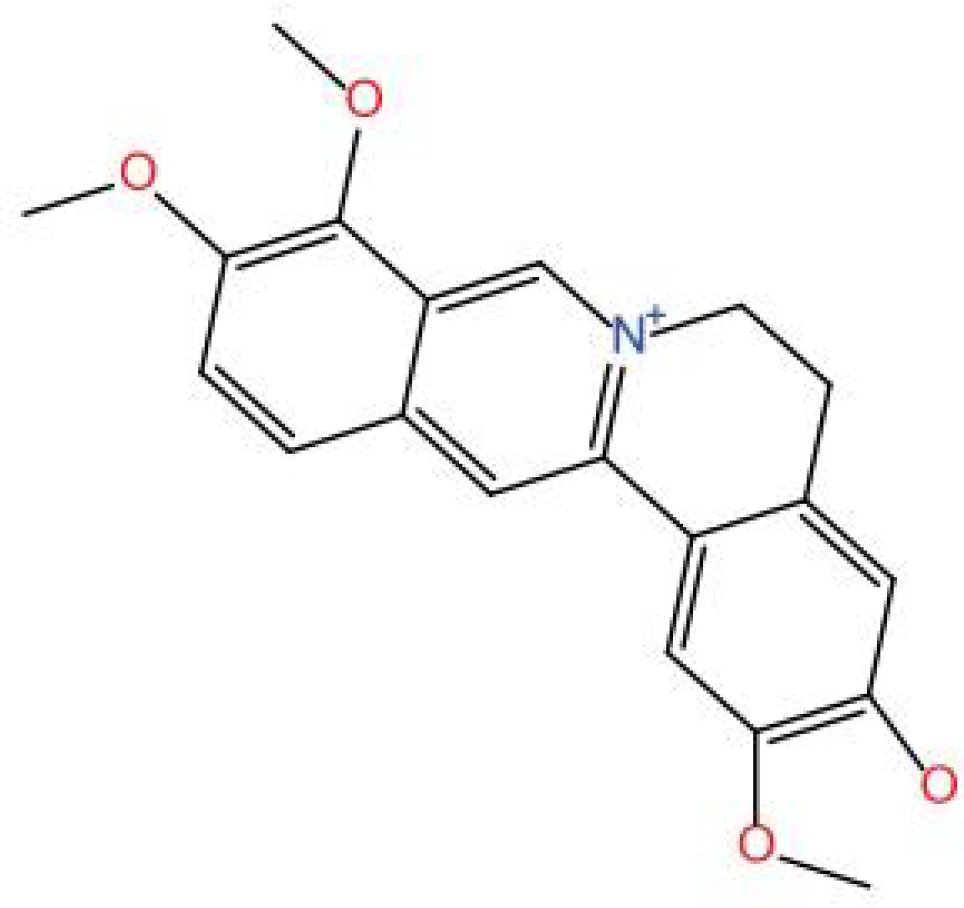	CORT mouse model	p.o 100,200 mg·kg^−1^ for 4 weeks	NLRP3 inflammasome-associated neuroimmune regulation and synaptic remodeling pathways	TNF-α; IL-1β; IL-6; NLRP3↓ BrdU;DCX; PSD-95;SYN↑	Associative evidence	(128)
Wogonin	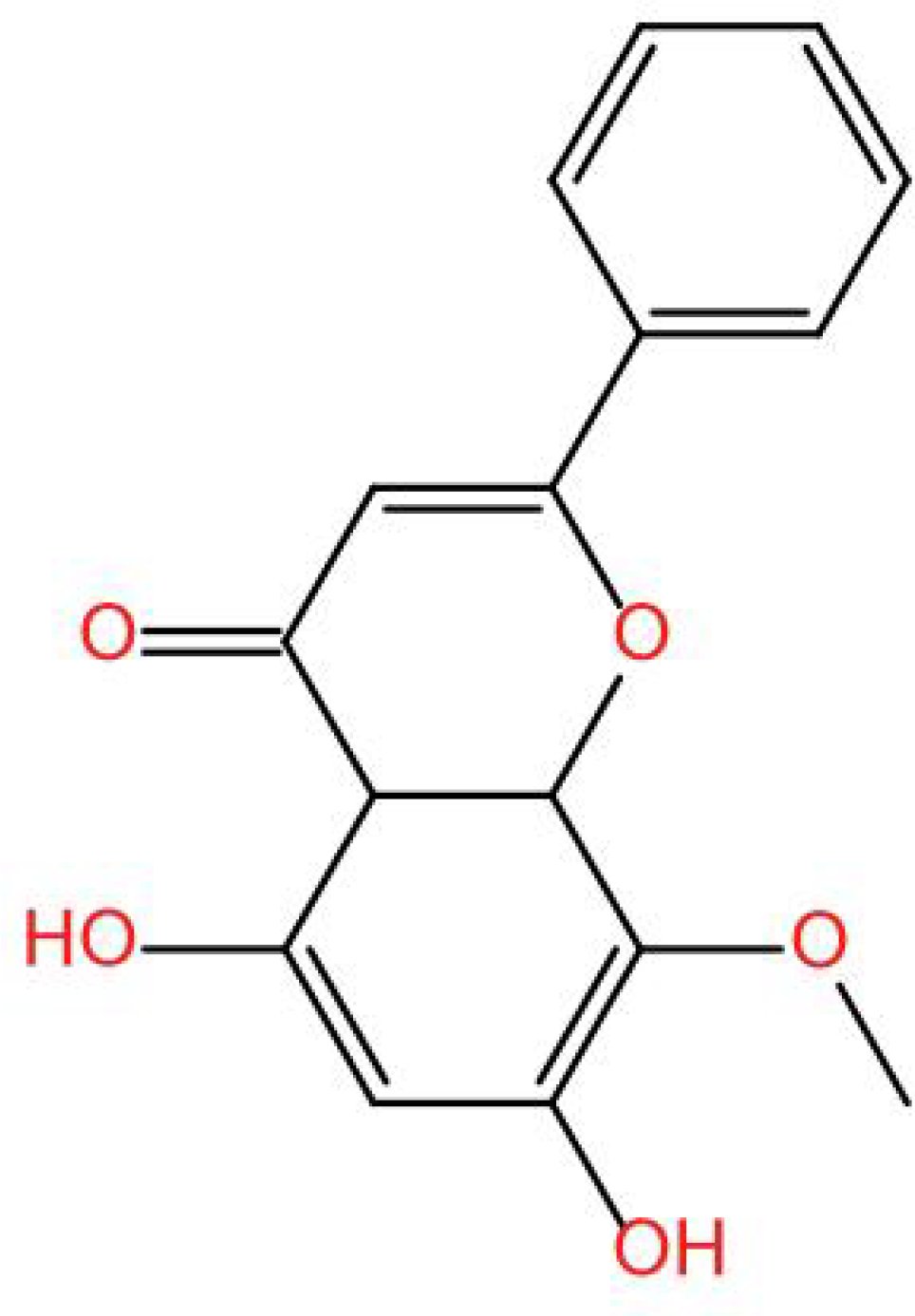	CUMS mouse model +LPS cell model	i.g. 40 mg·kg^-1^ for 2 weeks (mouse model); 10 μM (cell model)	Complement C3- and NLRP3 inflammasome-associated neuroimmune pathways involved in synaptic remodeling	TNF-α; NLRP3; CXCL1; GFAP;C3↓	Associative evidence	(129)
Mahoniae Caulis Alkaloids	Not specified	CORT mouse model	i.g. 2.5,5,10 mg·kg ^-1^ for 5 weeks	BDNF/CREB-associated neuronal resilience and inflammatory regulation pathways	BDNF; PRKACA; p-CREB/CREB;Cx43↑ NF-κB↓	Indirect evidence	(130)
Tanshinone I	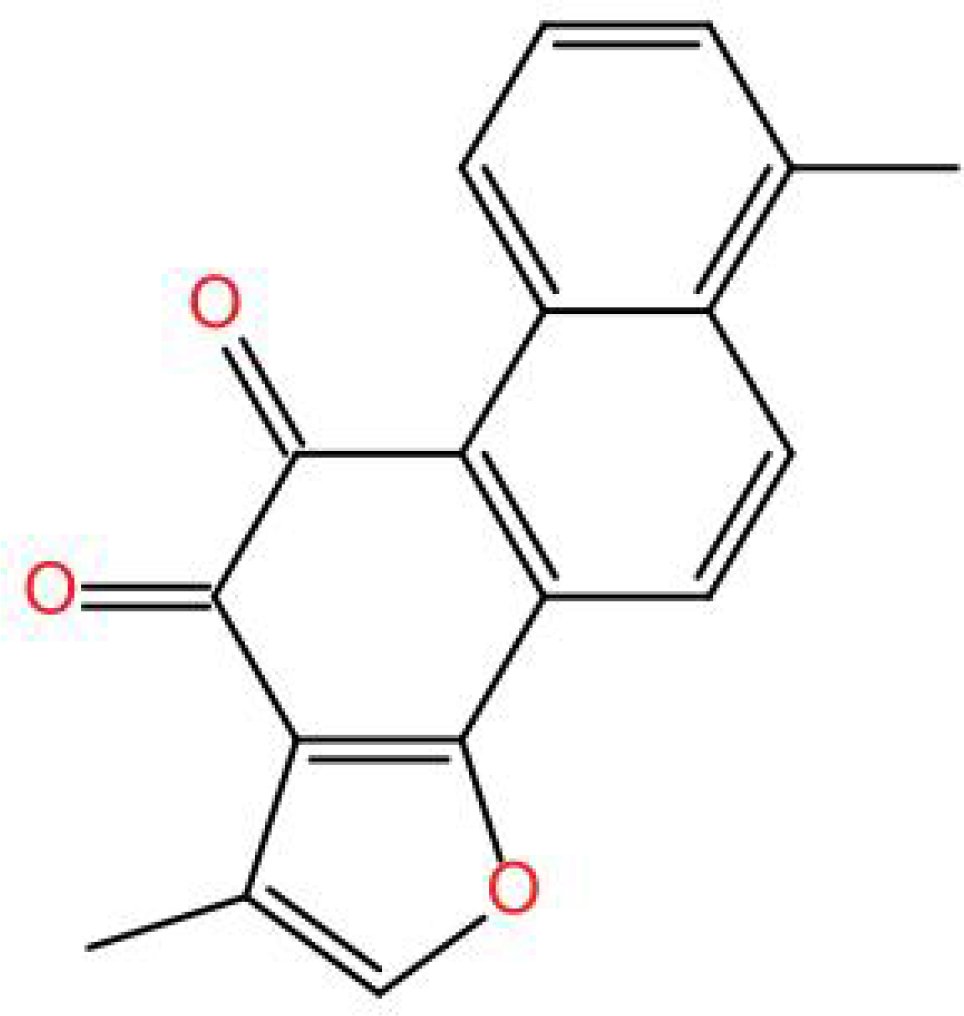	CUS mice model	i.g. 90 mg·kg ^-1^ sacrificed at intervals of 15, 30, 60, or 120 min	C3-associated complement pathway and STAT3-mediated neuroimmune regulation involved in synaptic remodeling	COX-2; iNOS; C3; p-STAT3 ↓ PSD-95;SYP↑	Associative evidence	(131)
Honokiol	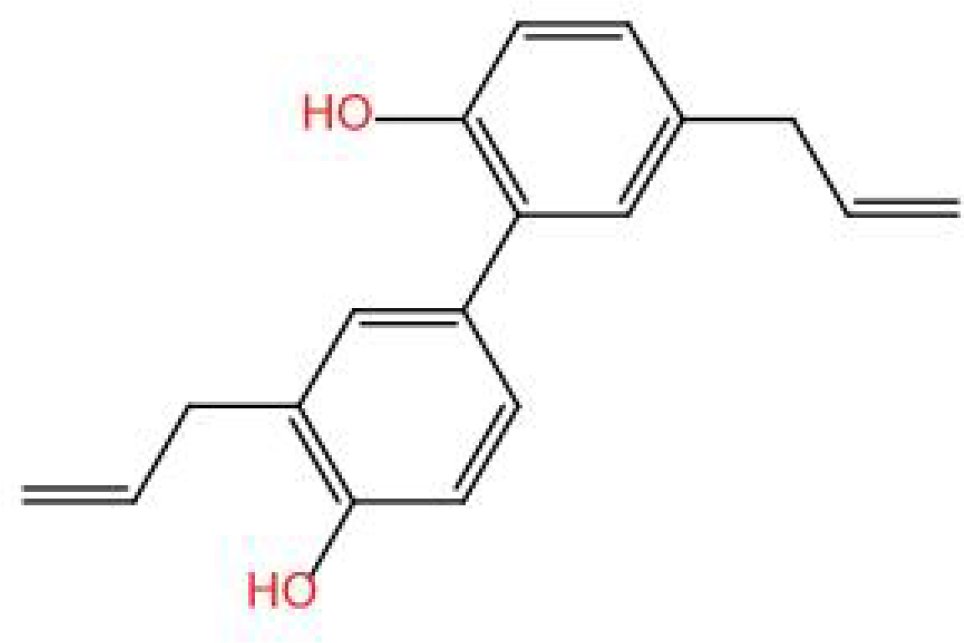	CUMS mice model	i.g. 10 mg·kg ^-1^ for 3 weeks	HIF-1α/VEGF-associated neurovascular and synaptic resilience pathways	HIF-1α;VEGF;VEGFR-2;SYN-1;PSD-95↑	Indirect evidence	(132)
Genipin 1-gentiobioside	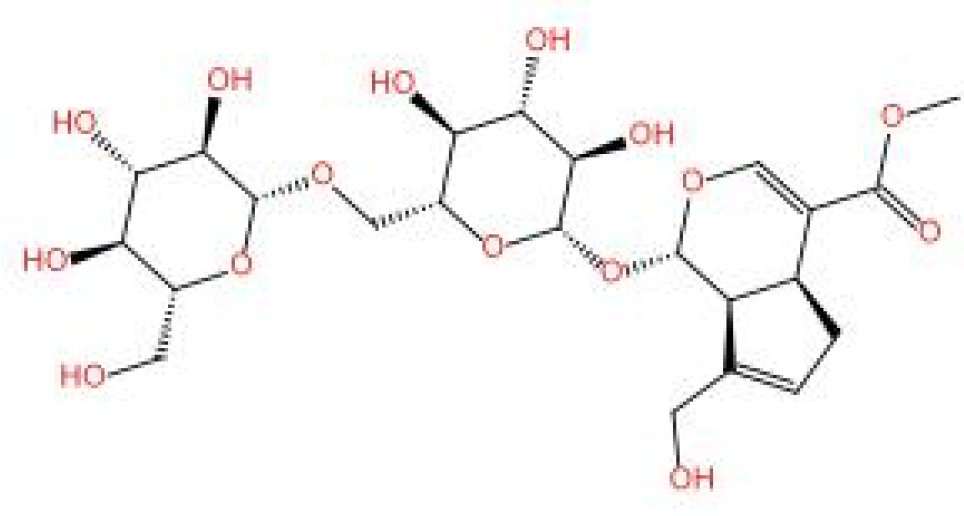	CUMS mice model	i.g. 25,50,100 mg·kg ^-1^ for 3 weeks	AMPA receptor-associated synaptic resilience and glutamatergic remodeling pathways	PICK1;GluA2;PSD-95↑	Indirect evidence	(133)
Loganin	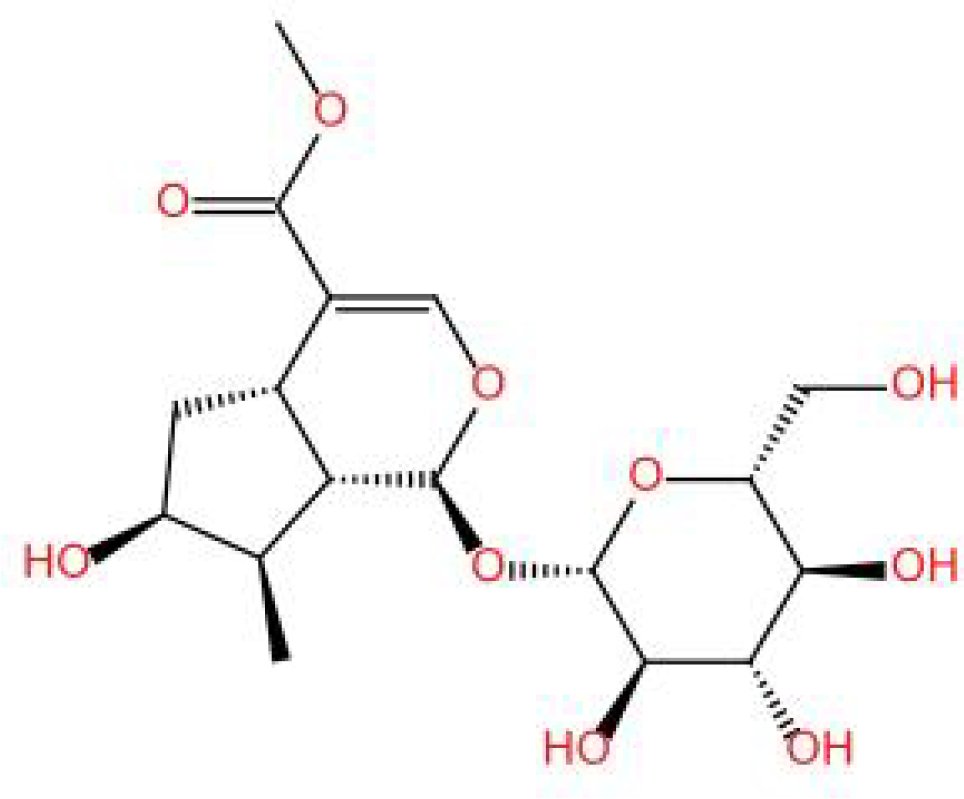	CUS rats model	i.g. 8.75,35 mg·kg ^-1^ for 4 weeks	Immune-inflammatory regulation pathways associated with neuronal resilience	iNOS; Th17; RORγt; IL-17 ↓ Arg-1↑	Indirect evidence	(134)
Jujuboside A	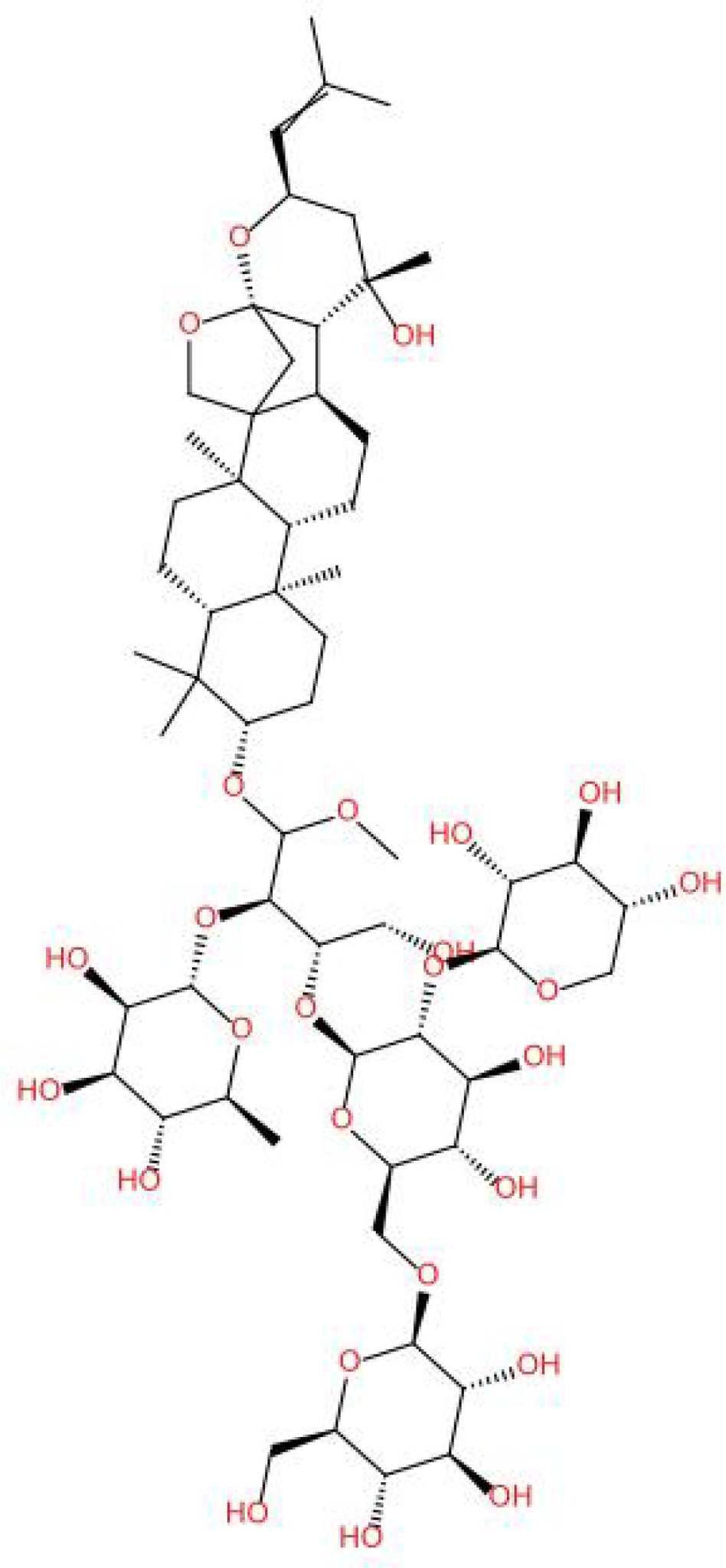	CUMS mice model	i.g. 12.5,25,50 mg·kg ^-1^ for 4 weeks	Synaptic resilience and neurogenesis-associated structural remodeling pathways	Syn;PSD-95↑ DCX/CaMKII↓	Indirect evidence	(135)
Paeoniflorin	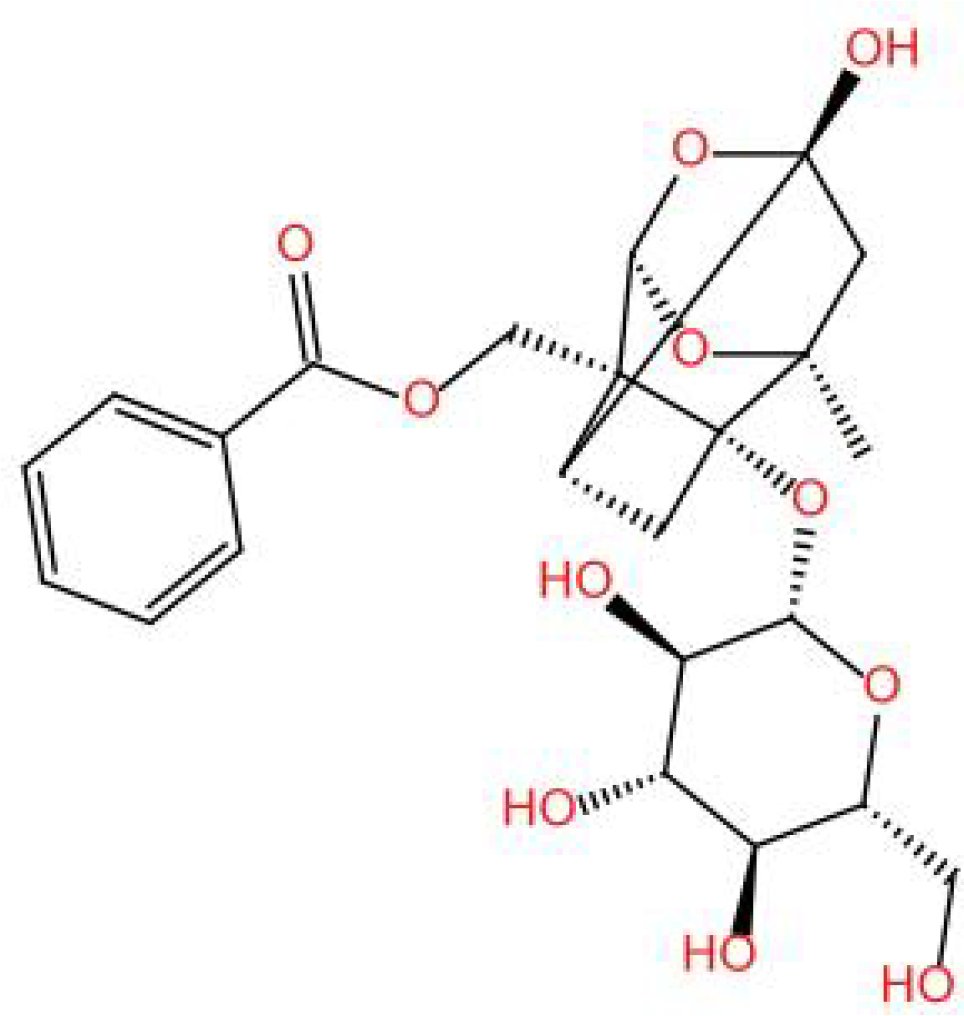	CUMS mice model	i.g. 20 mg·kg ^-1^ for 2 weeks	Inflammatory regulation pathways associated with neuronal resilience	IL-1β; TNF-α↓	Indirect evidence	(136)
Paeoniflorin	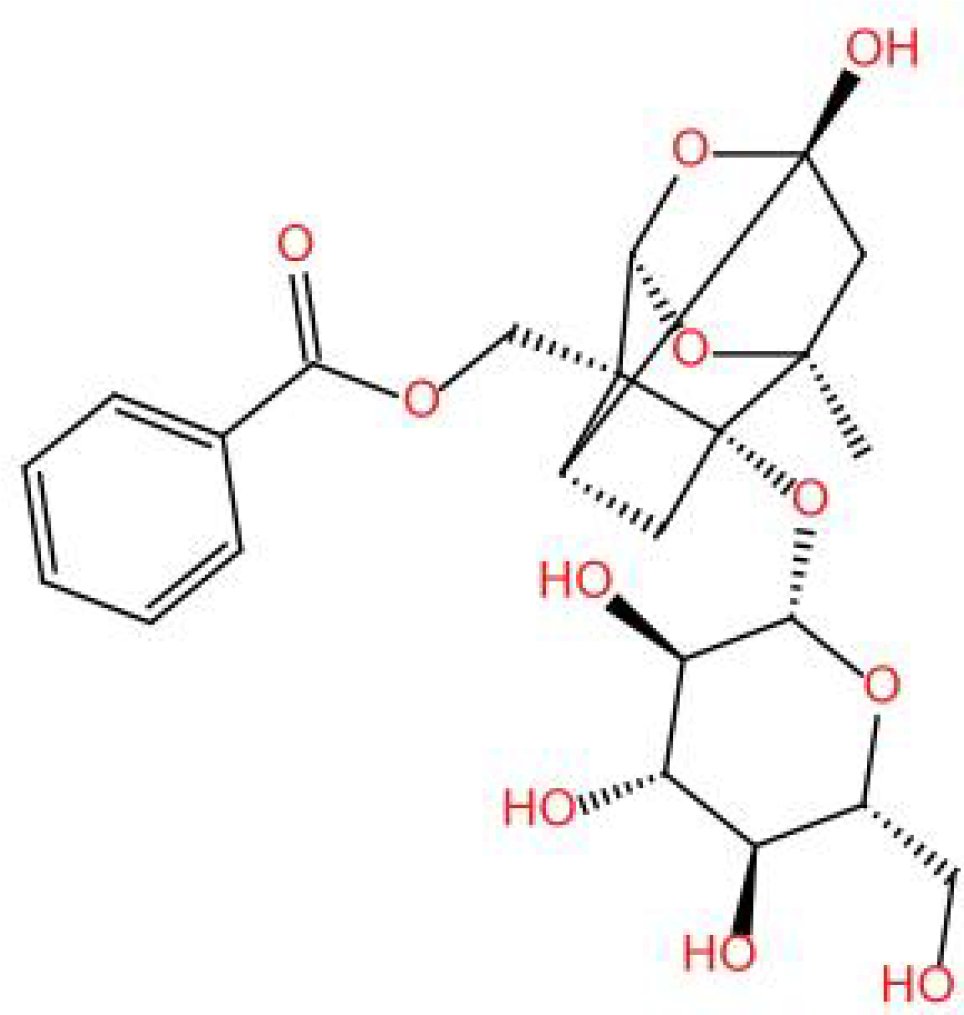	LPS-induced depression-like model	p.o. 20,40,80 mg·kg ^-1^ for 1 week	Iba1/TLR4-associated microglial inflammatory regulation and synaptic resilience pathways	BDNF;TrkB;PSD-95;SNAP25; NQO1;Nrf2;HO-1; p-ERK↑ Iba1; TNF-α;IL-1β;IL-6; GSK3β; Keap1; p-JNK;p-P38; TLR4↓	Associative evidence	(137)
*Schisandra chinensis* polysaccharide	Not specified	UC mice model	i.g. 200,800 mg·kg ^-1^ for 2 weeks	gut-brain axis-associated inflammatory regulation and synaptic resilience pathways	PSD-95; *Bacteroidales_bacterium*;*Dubosiella_muris*;*Lactobacillus_taiwanensis*;*Limosilactobacillus_reuteri*;*Allobaculum_mucilyticum*↑ N’-formylkynurenine;3-hydroxyanthranilic acid; pyruvic acid; NAD; NF-κB; TNF-α↓	Indirect evidence	(138)
Asperosaponin VI	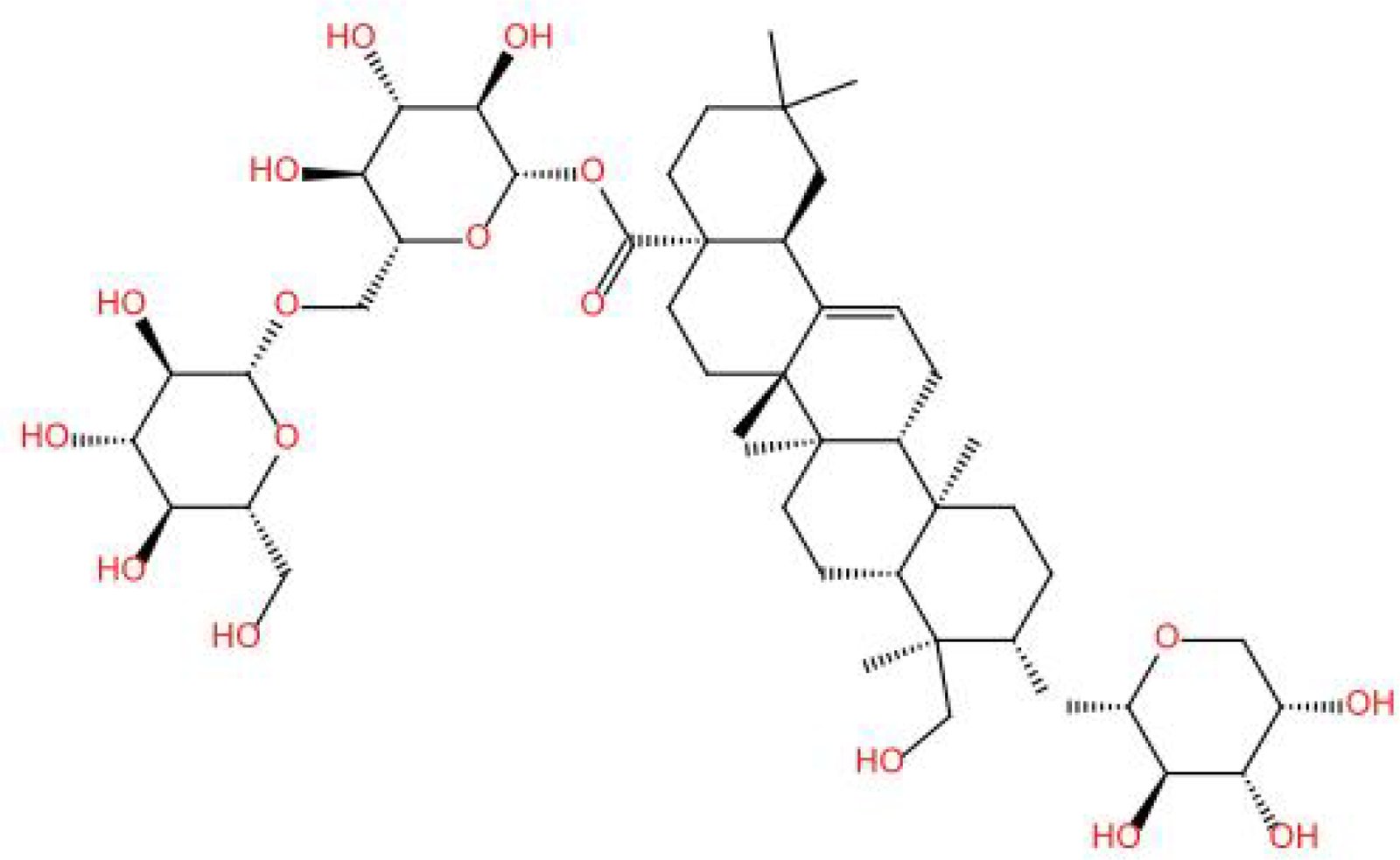	CMS mice model	i.g. 40 mg·kg ^-1^ for 1 week	Inflammatory regulation and BDNF-associated synaptic resilience pathways	iNOS; IL-1β;IL-6;TNF-α↓ Arg-1; IL-10; TGF-β; BDNF; PSD-95;CaMKIIα; CaMKIIβ; GluA2↑	Indirect evidence	(139)
Panacis Quinquefolii Radix Polysaccharides	Not specified	CUMS mice model	i.g. 500 mg·kg ^-1^ for 4 weeks	C1q/C3-associated complement pathway and gut-brain axis-mediated neuroimmune regulation	C1q;C3; *norank_f:Muribaculaceae;Alistipes;* TNF-α;IL-1β; IL-6↓ *Candidatus_Saccharimonas*;*norank_o:Clostridia_UCG-014*;*norank_f:Oscillospiraceae;* IL-10↑	Associative evidence	(140)

Overall, current evidence from single Chinese herbs mainly supports their ability to regulate glial activation, immune environments, and synaptic resilience. Although some studies involve pathways associated with microglial function, direct evidence demonstrating altered microglial synaptic engulfment remains limited.

#### 5.3.1 Enhancement of synaptic resilience through the BDNF/TrkB neurotrophic signaling pathway

The BDNF/TrkB pathway plays a critical role in maintaining synaptic survival, structural stability, and stress resilience. Its impairment in depression may increase neuronal vulnerability to stress-induced synaptic remodeling. Bioactive compounds derived from traditional Chinese medicinal sources may enhance this protective signaling network through activation of downstream pathways, including MAPK, PI3K, and mTOR, thereby promoting synaptic structural maintenance and neuronal resilience. Ginsenoside Rb1 activates the BDNF-TrkB signaling cascade by inhibiting miR-134 expression, which relieves its suppression of the BDNF-3’UTR. This mechanism enhances synaptic survival signaling and improves synaptic resilience under chronic stress conditions (118). Rosmarinic Acid (RA) exhibits a dual defense mechanism: on one hand, it upregulates the BDNF/TrkB/CREB and PI3K/Akt/mTOR pathways, increasing dendritic spine density in the hippocampal CA3 region and promoting neurogenesis in the dentate gyrus (DG); on the other hand, it mitigates the stress hormone-associated synaptic impairment by regulating glucocorticoid receptor (GR) nuclear translocation (reducing p-GR/GR, HSP90, etc.). LY294002 reversal experiments indicate that PI3K/Akt signaling contributes to the synaptic protective effects of RA (119). Engeletin specifically enhances the activity of the prefrontal cortical BDNF-TrkB-mTORC1 pathway under chronic restraint stress, reversing the reduction in dendritic spine density and reducing susceptibility to stress-associated synaptic impairment by supporting synaptic structural integrity (120). Lycopene and Kaempferol-3-O-sophoroside (PCS-1) induce neuronal proliferation and neurite outgrowth by activating the BDNF-TrkB/p-TrkB and BDNF/TrkB/Akt pathways, respectively. These effects may enhance synaptic structural resilience under inflammatory conditions (121, 122). Epigallocatechin gallate (EGCG) promotes BDNF expression by activating the CREB transcription factor. This not only facilitates neuronal development but also provides essential nutritional support for synapses, supporting synaptic stability under stress conditions (123). Baicalin and Quercetin focus on restoring synaptic protection within an anti-inflammatory context. Baicalin restores redox homeostasis and reduces ROS accumulation by activating the BDNF-TrkB pathway, reducing oxidative stress-associated synaptic impairment (124). Quercetin inhibits neuroinflammation via the PI3K/Akt pathway, improves synaptic ultrastructure, and improves inflammation-associated synaptic alterations (125).

Current evidence indicates that bioactive compounds derived from traditional medicinal sources mainly enhance synaptic resilience through neurotrophic and structural mechanisms. Although these pathways may influence susceptibility to pruning-related alterations, direct evidence demonstrating modulation of microglial synaptic engulfment remains lacking.

#### 5.3.2 Modulation of neuroinflammation and microglial activation states

Neuroinflammatory activation represents an important factor that may contribute to the transition of microglia from a homeostatic state toward reactive phenotypes associated with altered phagocytic activity. In depression, activated microglia may contribute to complement activation and increased synaptic vulnerability through the release of pro-inflammatory cytokines and regulation of complement-related proteins.

Regarding the targeting of complement and phagocytic receptors, Ginsenoside Rg3 provides a relatively direct link between bioactive compounds and pruning-associated complement signaling. It downregulates C1q expression, which may reduce complement-associated synaptic alterations and modulate microglial responses, thereby improving depressive-like behaviors in experimental models (126). In terms of inhibiting inflammasomes and cytokine storms, Emodin may reduce inflammatory and phagocytosis-associated microglial responses by regulating 5-LO and GSK3β signaling and blocking the release of IL-1β and TNF-α in the hippocampus, thereby improving neuronal integrity and dendritic spine alterations (127). Berberine attenuates neuroinflammatory responses by inhibiting activation of the NLRP3 inflammasome and blocking the inflammatory cascade, reducing inflammatory mediator-associated impairment of synaptic plasticity and neurogenesis (128). Concerning the regulation of chemokines and signaling pathways, Wogonin improves the microenvironment in the hippocampal CA1 region by modulating the TNF-α/TNFR1/CXCL1 signaling axis. This regulation may alleviate neuronal damage and restore synaptic functions, including long-term potentiation (LTP), thereby improving inflammation-associated synaptic dysfunction (129). Mahoniae Caulis Alkaloids (MA) mitigate neuroinflammation-associated alterations in synaptic structures by regulating cAMP signaling and gap junction pathways, thereby modulating glial cell communication and inflammatory responses (130). In the realm of pleiotropic anti-inflammatory protection, EGCG significantly reduces serum IL-1β levels while activating the BDNF/TrkB neurotrophic pathway. Through these combined neurotrophic and anti-inflammatory effects, EGCG may alleviate inflammation-associated synaptic impairment (123).

Based on the available evidence, bioactive compounds derived from traditional medicinal sources may regulate microglial activation states and inflammatory pathways associated with pruning susceptibility. Among these mechanisms, complement-related regulation, such as C1q modulation, provides relatively closer associative evidence linking these compounds to pruning-associated processes. However, direct demonstration of altered microglial synaptic engulfment remains limited.

#### 5.3.3 Regulation of synaptic structure and plasticity-related proteins associated with pruning susceptibility

The integrity of synaptic structure represents an important determinant of synaptic resilience under pathological conditions, and the stability of postsynaptic density proteins contributes to the maintenance of synaptic function. Certain bioactive compounds derived from traditional medicinal sources enhance synaptic structural stability through regulation of synaptic protein expression and may also influence complement-related signaling pathways involved in synaptic remodeling. These effects may influence complement-associated recognition processes and phagocytosis-related signaling involved in synaptic remodeling.

As a potent natural regulator of complement C3, Tanshinone I has been reported to regulate complement-associated signaling through modulation of C3-related pathways. By interacting with C3 protein, Tanshinone I may attenuate C3-CR3 axis activation and downstream STAT3 signaling, thereby potentially modulating microglia-associated synaptic remodeling in experimental models (131). Honokiol significantly promotes the expression of SYN1 and PSD-95 proteins both *in vivo* and *in vitro* by activating the HIF-1α-VEGF signaling pathway. This upregulation of synaptic structural proteins may enhance synaptic stability and resilience under pathological stress conditions (132). Geniposide improves the structural integrity of hippocampal synapses in CUMS mice and ameliorates synaptic structural alterations by specifically binding to the PICK1 protein and regulating the expression levels of synaptic plasticity-related proteins (133). Loganin intervenes in synaptic fragility caused by HINT1 overexpression. Since HINT1 typically interferes with the interaction of Sigma-1R to NMDAR and inhibits BDNF signaling-leading to impaired synaptic plasticity-Loganin restores postsynaptic receptor-associated signaling by modulating this process, thereby maintaining synaptic structural stability (134). Jujuboside A regulates calcium homeostasis in immature neurons via the Shh signaling pathway, optimizing the plasticity of synaptic structure. These effects may support the stability of newly formed synapses within complex microenvironments and reduce susceptibility to calcium imbalance-associated structural alterations (135).

Among these compounds, C3-related modulation by Tanshinone I provides relatively closer associative evidence linking bioactive compounds with complement-associated pruning mechanisms. However, most compounds described in this section primarily demonstrate indirect effects through regulation of synaptic proteins and neuronal structural resilience, and direct evidence of altered microglial synaptic engulfment remains limited.

#### 5.3.4 Multi-pathway regulation of synaptic resilience and pruning-associated processes

Through their multi-target and multi-pathway properties, bioactive compounds derived from traditional Chinese medicinal sources may influence regulatory processes spanning peripheral homeostasis, central neuroimmune responses, and synaptic structural maintenance. By regulating gut microenvironmental homeostasis, inflammatory responses, and neurotrophic support, these compounds may influence microglia-associated processes related to synaptic remodeling and enhance overall synaptic resilience.

Paeoniflorin exhibits multi-pathway protective effects. On one hand, it inhibits NLRP3 inflammasome activation via the Akt/GSK3β pathway and suppresses TLR4/NF-κB signaling, reducing the release of pro-inflammatory cytokines such as TNF-α and IL-1β, thereby attenuating inflammatory signals associated with synaptic vulnerability. On the other hand, it enhances the Nrf2/HO-1 antioxidant axis, restores mitochondrial membrane potential, and reduces oxidative stress-associated synaptic damage. Building on this foundation, Paeoniflorin upregulates the expression of PSD-95, SNAP25, and BDNF in the hippocampus and increases dendritic spine density. Through this triple mechanism involving anti-inflammatory, antioxidant, and neurotrophic effects, Paeoniflorin improves LPS-induced synaptic alterations (136, 137). Schisandra chinensis Polysaccharide focuses on protecting synapses through bidirectional regulation of the “gut-brain axis.” It reduces systemic and central inflammatory loads by repairing intestinal barrier integrity and alleviating colonic inflammation. This restoration of peripheral immune homeostasis may indirectly alleviate neuroinflammation-associated hippocampal neuronal impairment, support synaptic plasticity, and reduce susceptibility to inflammation-associated synaptic dysfunction (138). Asperosaponin VI has been reported to promote a shift of microglial phenotype toward a more homeostatic state through activation of the PPAR-γ signaling pathway. This phenotypic switching not only inhibits the release of inflammatory factors but also promotes the secretion of neurotrophic factors, improving chronic stress-associated hippocampal synaptic dysfunction and providing a microenvironment supportive of synaptic maintenance (139). Panacis Quinquefolii Radix Polysaccharides exerts antidepressant effects by suppressing complement-associated microglial synaptic pruning and restoring gut microbiota homeostasis through the gut-brain axis, thereby preserving excitatory synapses and alleviating CUMS-induced depressive-like behaviors (140).

Taken together, bioactive compounds derived from traditional medicinal sources may regulate multiple biological processes related to synaptic resilience, neuroinflammation, and microglial states. However, except for limited complement-related findings, direct evidence demonstrating that these compounds alter microglial synaptic engulfment remains insufficient.

## 6 Conclusions and prospects

In summary, dysregulated microglia-mediated synaptic pruning represents a critical link between pathological factors associated with depression, including chronic stress, neuroinflammation, metabolic disturbances, and impaired synaptic plasticity. Accumulating evidence indicates that neurotrophic deficits, inflammatory activation, mitochondrial dysfunction, and gut microbiota alterations may drive microglia from homeostatic states toward reactive phenotypes with altered phagocytic properties, thereby contributing to aberrant synaptic remodeling and impaired neural circuit function.

With its multi-component and multi-target characteristics, Traditional Chinese Medicine (TCM) represents a promising approach for modulating complex neuroimmune–synaptic interactions in depression. Current studies suggest that TCM formulas, herbal extracts, and bioactive compounds may influence biological processes associated with microglia–synapse communication, including neurotrophic support, complement-associated signaling, inflammatory regulation, mitochondrial homeostasis, and synaptic resilience. However, it should be noted that most existing studies primarily provide indirect or associative evidence, whereas direct evidence demonstrating TCM-mediated regulation of microglial synaptic pruning remains limited.

By integrating current knowledge of microglia-mediated synaptic pruning with evidence from TCM-related studies, this review provides an evidence-based framework for understanding how TCM interventions may influence pruning-associated mechanisms at the microglia–synapse interface. The current limitations also highlight important research opportunities. Future investigations incorporating single-cell sequencing, spatial transcriptomics, advanced imaging, molecular tracing, and functional phagocytosis assays are required to establish causal relationships between TCM interventions and microglia-mediated synaptic remodeling. Furthermore, exploration of the gut–brain axis, biomarker-guided approaches, and precision medicine strategies may facilitate a deeper understanding of individualized TCM-based interventions for depression.

Overall, targeting microglia-associated synaptic remodeling provides a valuable conceptual framework for elucidating depression pathophysiology and advancing the mechanistic understanding of TCM-based approaches. Emerging technologies in neuroimaging, systems biology, and precision medicine will further contribute to the development of evidence-based strategies for investigating the role of TCM in neuroimmune regulation and synaptic homeostasis.
